# Physical activity matters for everyone’s health, but individuals with multimorbidity benefit more

**DOI:** 10.1016/j.pmedr.2023.102265

**Published:** 2023-06-02

**Authors:** Layan Fessler, Silvio Maltagliati, Stefan Sieber, Stéphane Cullati, Elena Tessitore, Cecilia Craviari, Christophe Luthy, Eliana Hanna, Philippe Meyer, Dan Orsholits, Philippe Sarrazin, Boris Cheval

**Affiliations:** a Univ. Grenoble-Alpes, SENS, F-38000 Grenoble, France; b LIVES Centre, Swiss Centre of Expertise in Life Course Research, University of Lausanne, Switzerland; c Population Health Laboratory (#PopHealthLab), University of Fribourg, Switzerland; d Department of Readaptation and Geriatrics, University of Geneva, Switzerland; e Department of Cardiology and Department of Internal Medicine and Rehabilitation, University Hospital of Geneva, Switzerland; f Department of Movement, Human and Health Sciences, University of Rome “Foro Italico”, Rome, Italy; g Unit of Internal Medicine and Rehabilitation, Department of Rehabilitation and Geriatrics, Geneva University Hospital, Geneva, Switzerland; h Division of General Medical Rehabilitation, University Hospitals of Geneva, Switzerland; i Cardiology Service, Geneva University Hospitals, Geneva, Switzerland; j Swiss NCCR “LIVES – Overcoming Vulnerability: Life Course Perspectives”, University of Geneva, Geneva, Switzerland; k Swiss Center for Affective Sciences, University of Geneva, Switzerland; l Laboratory for the Study of Emotion Elicitation and Expression (E3Lab), Department of Psychology, University of Geneva, Switzerland

**Keywords:** Multimorbidity, Chronic conditions, Physical activity, Health, Aging, Europe

## Abstract

Multimorbidity, defined as the presence of two or more chronic conditions, is increasingly prevalent and is a major contributor to ill health in old age. Physical activity (PA) is a key protective factor for health and individuals with multimorbidity could particularly benefit from engaging in PA. However, direct evidence that PA has greater health benefits in people with multimorbidity is lacking. The objective of the present study was to investigate whether the associations between PA and health were more pronounced in individuals with (vs. without) multimorbidity. We used data from 121,875 adults aged 50 to 96 years (mean age = 67 ± 10 years, 55% women) enrolled in the Survey of Health, Ageing and Retirement in Europe (SHARE). Multimorbidity and PA were self-reported. Health indicators were assessed using tests and validated scales. Variables were measured up to seven times over a 15-year period. Confounder-adjusted linear mixed-effects models were used to investigate the moderating role of multimorbidity on the associations of PA with the levels and trajectories of health indicators across aging. Results showed that multimorbidity was associated with declines in physical, cognitive, and mental health, as well as poorer general health. Conversely, PA was positively associated with these health indicators. We found a significant interaction between multimorbidity and PA, revealing that positive associations between PA and health indicators were strengthened in people with multimorbidity – although this stronger association became less pronounced in advanced age. These findings suggest that the protective role of PA for multiple health indicators is enhanced in individuals with multimorbidity.

## 1 Introduction

Human life expectancy, especially in higher-income countries (Kirkwood, 2017), has increased rapidly in the recent history of human evolution. However, healthy life expectancy has not followed the same pace (Salomon et al., 2012). Multimorbidity, defined as the presence of two or more chronic conditions (Duggal et al., 2019), is increasingly prevalent, affecting more than 50% of adults aged 50 years or older (Xu et al., 2017). Crucially, multimorbidity is a major contributor to ill health in old age (Duggal et al., 2019). Multimorbidity is associated with poorer physical (Wei et al., 2018) and cognitive health (Loprinzi, 2016, Quiñones et al., 2022), poorer health-related quality of life (Makovski et al., 2019), as well as weaker mental (Jones et al., 2016) and self-rated health (Marques et al., 2018). Due to the global increase in individuals with multimorbidity in the context of an aging population (Kingston et al., 2018), developing interventions for managing multimorbidity is urgently needed. While massive efforts have been made to improve pharmacological treatments (Dumbreck et al., 2015, Vetrano et al., 2020), less attention has been paid to non-pharmacological treatments such as physical activity (PA).

PA, defined as any bodily movement produced by skeletal muscles that results in energy expenditure (Caspersen et al., 1985), has the potential to be particularly effective in buffering the detrimental effects of multimorbidity on health, all-cause mortality, and life expectancy (Chudasama et al., 2019, Loprinzi et al., 2016, Marques et al., 2018, Martinez-Gomez et al., 2017). Studies have shown that multimorbidity is generally associated with systemic inflammation, in part due to age-related sarcopenia (Duggal et al., 2019). In turn, this systemic inflammation may explain the alterations in physical, cognitive, and mental health observed in individuals with multimorbidity (Chu et al., 2021, Gleeson et al., 2011, Trollor et al., 2012). Importantly, PA has been found to reduce systemic inflammation associated with age-related multimorbidity through its beneficial effects on sarcopenia and immunosenescence (Dalle et al., 2017, Duggal et al., 2019). Accordingly, due to such anti-inflammatory effects, individuals with (vs. without) multimorbidity may particularly benefit from PA. Yet, direct evidence showing that PA has more pronounced health benefits among individuals with (vs. without) multimorbidity is lacking. Whether this stronger protective role occurs across multiple health indicators remains unknown. Finally, little is known about whether and how age might moderate the strength of these associations.

The objective of the present large-scale longitudinal study was to examine whether the positive associations between PA and multiple health indicators (i.e., physical, mental, cognitive, and general health) were stronger in individuals with (vs. without) multimorbidity. We hypothesized that PA (H1) and multimorbidity (H2) would be positively and negatively associated with health, respectively. Furthermore, because the protective role of PA is thought to be particularly relevant in the context of systemic inflammation associated with multimorbidity (Duggal et al., 2019), we hypothesized that the positive associations between PA and health indicators would be stronger in individuals with (vs. without) multimorbidity (H3). Finally, we investigated the association of PA and multimorbidity with the trajectories of the health indicators across aging. We hypothesized that PA (H4) and multimorbidity (H5) would be associated with a weaker and stronger decline in the health indicators across aging, respectively. We hypothesized that the protective role of PA in slowing age-related health decline would be particularly pronounced among individuals with (vs. without) multimorbidity (H6).

## 2 Methods

### 2.1 Study design

Data were drawn from the Survey of Health, Ageing and Retirement in Europe (SHARE), a longitudinal population-based study of adults aged 50 years and older living in 28 European countries and Israel (Börsch-Supan et al., 2013). Data were collected using computer-assisted personal interviewing (CAPI) in participants’ homes every 2 years between 2004 and 2020 (8 waves of data collection). Chronic conditions, PA, and health indicators were assessed repeatedly across waves of measurement, with the exception of wave 3, which was dedicated to data collection on childhood history (SHARELIFE). Thus, chronic conditions and PA were treated as time-varying predictors. As such, participants who developed multimorbidity during the follow-up were then coded as having multimorbidity, whereas they were coded as having no-multimorbidity at baseline. SHARE was conducted in accordance with the Declaration of Helsinki and was approved by the Ethics Committee of the University of Mannheim for waves 1 to 4 and by the Ethics Council of the Max Planck Society for waves 4 to 8. All participants provided a written informed consent. Inclusion criteria were as follows: 1) to be between 50 and 96 years of age, 2) to have completed at least one item on the Chronic Disease Questionnaire, and 3) on the PA Questionnaire, and 4) to have reported at least one health indicator.

### 2.2 Measures

#### 2.2.1 Outcomes: physical, cognitive, mental, and general health indicators

All the measures are described in detail in Supplemental materials 1.

*Physical health* was assessed through maximal grip strength using a handheld dynamometer (Smedley, S Dynamometer, TTM, Tokyo, 100 kg) (Cheval et al., 2018a, Leong et al., 2015).

*Cognitive health* was measured using the 10-words delayed recall test (Harris and Dowson, 1982).

*Mental health* was measured by two indicators: depressive symptoms using the EURO-D scale (Prince et al., 1999), and well-being using the short version of the CASP-19 (Hyde et al., 2003).

*General health* was assessed using the self-rated health questionnaire (Ware and Sherbourne, 1992).

#### 2.2.2 Exposures: multimorbidity and PA

*Multimorbidity.* Chronic conditions were assessed using the following question: “Has a doctor ever told you that you had / Do you currently have any of the conditions on this card?”. The card contained a list of 18 conditions. We defined multimorbidity as the coexistence of ≥ 2 chronic conditions (Johnston et al., 2019), with individuals being classified as either having or not having multimorbidity.

*PA* was derived from the following two questions: “*How often do you engage in activities that require a low or moderate level of energy such as gardening, cleaning the car, or going for a walk?*” and “*How often do you engage in vigorous PA, such as sports, heavy housework, or a job that involves physical labor?*” (Cheval et al., 2020, Cheval et al., 2019, de Souto Barreto et al., 2017). Participants were classified as either physically active or inactive.

**Potential confounders.** Confounders were identified using directed acyclic graphs. The identified confounders were: the wave of measurement, the sex, the birth cohort, attrition, the country of residence, the ability to make ends meet, and education.

### 2.3 Statistical analysis

Descriptive statistics stratified by multimorbidity status were calculated. We then used linear mixed-effects models to examine the independent associations of PA and multimorbidity with the different health indicators, as well as to test whether these associations between PA and the different health indicators were stronger in individuals with (vs. without) multimorbidity (i.e., moderation). By accounting for the nested structure of the data (i.e., repeated measures over time for a single participant), mixed-effects models provide acceptable type I error rates (Boisgontier and Cheval, 2016). Furthermore, because mixed-effects models do not require an equal number of observations across participants, those with missing observations could also be included (Raudenbush and Bryk, 2002). Thus, even participants with one wave of measurement were included in the models. Statistical analyses were conducted using the R packages lme4 and lmerTest (Bates et al., 2015, Kuznetsova et al., 2016, R Core Team, 2017). Effect size estimates for fixed effects were reported using marginal pseudo-R^2^ calculated using the MuMin package (Barton, 2018).

The modeling strategy was similar for all five health indicators. Model 1a tested the independent associations of multimorbidity and PA with the level of the health indicators, adjusting for prior confounders. Model 1b included interaction terms between multimorbidity and age (linear and quadratic), and between PA and age (linear and quadratic) to test whether multimorbidity and PA were independently associated with the trajectories of the health indicators across aging. Age was centered around the sample median (73 years). The quadratic effect of age was included to account for the potential accelerated rate of change in health indicators across aging. Model 2a tested the interaction between multimorbidity and PA at the level of health indicators. To do so, an interaction between multimorbidity and PA was added – a significant interaction indicating that multimorbidity significantly moderated the strength of the association between PA and the level of health indicators. Furthermore, to examine whether multimorbidity moderates the association between PA and health trajectories across aging, a three-way interaction between multimorbidity, PA, and age (linear and quadratic) was included in Model 2b – a significant interaction indicating that multimorbidity significantly moderates the association between PA and health trajectories across aging. The models’ random structure encompassed random intercepts for participants and random linear trajectories for age. This allows the participants to have their own (linear) evolution of their health indicators across aging. The quadratic effect of age was not added in the random structure due to convergence issues. Minimally-adjusted models were also fitted by only including wave of measurement, age, and sex as confounders (see Supplementary Tables S2 to S6).

## 3 Results

### 3.1 Descriptive statistics

Table 1 and Supplemental Table S1 summarize the characteristics of the participants stratified by multimorbidity status at baseline. The study sample consisted of 121,875 individuals aged 50 to 96 years (67 ± 10 years old, 55% women), 71% were classified as physically active and 56% with multimorbidity.

**Table 1:** Participants’ characteristics by multimorbidity status at baseline.

**N = 121,875**	**No multimorbidity** (N = 53,997)		**Multimorbidity** (N = 67,878)		***P* value**
**Variables**					
**Physical activity** (*n*; *%*)					
No	12,443	23.04	22,766	33.54	
Yes	41,554	76.96	45,112	66.46	<0.001
Age at baseline (*years; SD*)	61.60	9.248	65.915	9.797	<0.001
**Confounders**					
Sex (*n*; *%*)					
Female	2,8165	52.16	3,8967	57.41	
Male	2,5832	47.84	2,8911	42.59	<0.001
Birth cohort (*n*; *%*)					
After 1945	35,440	65.63	32,530	47.92	
Between 1919 and 1928	2,422	4.49	5,735	8.45	
Between 1929 and 1938	7,039	13.04	15,313	22.56	
Between 1939 and 1945	9,096	16.85	14,300	21.07	<0.001
Attrition (*n*; *%*)					
No drop out	31,146	57.68	37,274	54.91	
Drop out	18,833	34.88	20,740	30.55	
Death	4,018	7.44	9,864	14.53	<0.001
Able to make ends meet (*n*; *%*)					
Easily	21,604	40.01	20,201	29.76	
Fairly easily	16,697	30.92	20,481	30.17	
With some difficulties	10,614	19.66	16,977	25.01	
With great difficulties	5,082	9.41	10,219	15.06	<0.001
Education (*n*; *%*)					
Primary	9,663	17.89	18,459	27.19	
Secondary	30,420	56.34	37,720	55.57	
Tertiary	13,914	25.77	11,699	17.24	<0.001
**Outcomes** (*mean; SD*)					
Physical health					
Maximal muscle strength	36.19	11.79	32.41	12.25	<0.001
Cognitive health					
Delayed recall	2.61	1.42	2.99	1.30	<0.001
Mental health					
Depressive symptoms	1.82	1.93	3.01	2.45	<0.001
Well-being	38.7	5.77	35.55	6.51	<0.001
General health					
Self-rated health	3.39	1.01	2.45	0.98	<0.001

**Notes.** Baseline = the first measurement occasion for each participant; SD = standard deviation, *p* values were based on the analysis of variance and chi-square tests for continuous and categorical variables, respectively, testing the effect of multimorbidity status at baseline (multimorbidity vs. no multimorbidity) on these variables. Ability to make ends meet refers to pay for the things that individuals need to live when they have little money. The descriptive statistics were based on the larger sample size (i.e., 121,875 from the models testing depressive symptoms).

### 3.2 Independent associations of PA and multimorbidity with the levels and trajectories of the health indicators

Table 2 and supplemental Tables S2 to S6 report the independent associations of PA and multimorbidity with the level and trajectories of the five health indicators.

**Table 2:** Independent and interactive effect of physical activity and multimorbidity with the levels and changes in health indicators.

	**Physical health**		**Cognitive health**		**Mental health**				**General health**	
	Maximal muscle strength		Delayed recall		Depressive symptoms		Well-being		Self-rated health	
	**Model 1a**	**Model 2a**	**Model 1a**	**Model 2a**	**Model 1a**	**Model 2a**	**Model 1a**	**Model 2a**	**Model 1a**	**Model 2a**
**Variables**	b (95% CI)	b (95% CI)	b (95% CI)	b (95% CI)	b (95% CI)	b (95% CI)	b (95% CI)	b (95% CI)	b (95% CI)	b (95% CI)
**Level**										
Physical activity (ref. no)										
YES	1.02 (0.97; 1.07)^***^	0.87 (0.79; 0.95)^***^	0.15 (0.13; 0.16)^***^	0.13 (0.11; 0.14)^***^	−0.51 (-0.53; −0.49)^***^	−0.34 (-0.36; −0.31)^***^	1.70 (1.66; 1.74)^***^	1.48 (1.42; 1.55)^***^	0.27 (0.27; 0.28)^***^	0.26 (0.25; 0.27)^***^
Multimorbidity (ref. no)										
YES	−0.56 (−0.61; −0.51)^***^	−0.74 (−0.83; −0.65)^***^	−0.05 (−0.06; −0.04)^***^	−0.07 (−0.09; −0.05)^***^	0.64 (0.63; 0.66)^***^	0.85 (0.82; 0.88)^***^	−1.38 (−1.42; −1.34)^***^	−1.64 (−1.71; −1.57)^***^	−0.50 (−0.50; −0.49)^***^	−0.51 (−0.52; −0.50)^***^
Physical activity (ref. no) × Multimorbidity (ref. no)										
YES		0.25 (0.15; 0.34)^***^		0.03 (0.01; 0.05)^**^		−0.28 (−0.31; −0.25)^***^		0.36 (0.28; 0.44)^***^		0.02 (0.01; 0.03)^**^
*R^2^*m	0.62	0.62	0.28	0.0.28	0.17	0.17	0.33	0.33	0.31	0.31
*R*^2^c	0.86	0.86	0.55	0.55	0.52	0.52	0.64	0.64	0.60	0.60

	**Model 1b**	**Model 2b**	**Model 1b**	**Model 2b**	**Model 1b**	**Model 2b**	**Model 1b**	**Model 2b**	**Model 1b**	**Model 2b**

**Rate of change**										
Age (ref. 73 years old)	−4.51 (−4.63; −4.39)^***^	−4.50 (−4.64; −4.37)^***^	−0.59 (−0.61; −0.57)^***^	−0.61 (−0.64; −0.59)^***^	0.39 (0.35; 0.42)^***^	0.40 (0.36; 0.44)^***^	−1.37 (−1.46; −1.28)^***^	−1.41 (−1.51; −1.32)^***^	−0.29 (−0.30; −0.27)^***^	−0.30 (−0.31; −0.28)^***^
Age (ref. 73 years old) squared	−0.53 (−0.58; −0.48)^***^	−0.49 (−0.56; −0.43)^***^	−0.13 (−0.14; −0.12)^***^	−0.14 (−0.15; −0.13)^***^	0.15 (0.13; 0.16)^***^	0.17 (0.15; 0.19)^***^	−0.40 (−0.44; −0.36)^***^	−0.44 (−0.49; −0.39)^***^	−0.02 (−0.03; −0.02)^***^	−0.03 (−0.04; −0.02)^***^
Age (ref. 73 years old) × Physical activity (ref. no)										
YES	−0.001 (−0.07; 0.06)	−0.02 (−0.13; 0.08)	0.01 (−0.004; 0.02)	0.04 (0.02; 0.06)^***^	−0.13 (−0.13; −0.10)^***^	−0.13 (−0.17; −0.10)^***^	0.34 (0.28; 0.39)^***^	0.39 (0.31; 0.48)^***^	0.05 (0.04; 0.05)^***^	0.07 (0.05; 0.08)^***^
Age (ref. 73 years old) squared × Physical activity (ref. no)										
YES	−0.04 (−0.09; 0.01)	−0.09 (−0.16; −0.02)*	−0.01 (−0.02; −0.002)*	0.003 (−0.01; 0.02)	0.003 (−0.01; 0.02)	−0.02 (−0.04; 0.001)	−0.04 (−0.08; −0.001)*	0.01 (−0.04; 0.07)	−0.002 (−0.01; 0.004)	0.002 (−0.01; 0.011)
Age (ref. 73 years old) × Multimorbidity (ref. no)										
YES	0.07 (0.01; 0.14)*	0.09 (−0.01; 0.20)	0.02 (0.01; 0.04)^***^	0.06 (0.04; 0.08)^***^	−0.003 (−0.02; 0.02)	−0.04 (−0.08; −0.01)^**^	−0.05 (−0.09; 0.02)	0.05 (−0.03; 0.13)	0.07 (0.06; 0.08)^***^	0.09 (0.08; 0.10)^***^
Age (ref. 73 years old) squared × Multimorbidity (ref. no)										
YES	0.07 (0.02; 0.11)^**^	−0.000 (−0.08; 0.08)	0.02 (0.01; 0.03)^***^	0.03 (0.02; 0.05)^***^	0.05 (0.04; 0.06)^***^	0.02 (0.00; 0.05)*	−0.07 (−0.10; −0.03)^***^	−0.02 (−0.08; 0.04)	−0.004 (−0.01; 0.002)	−0.004 (−0.01; 0.01)
Age (ref. 73 years old) × Physical activity (ref. no) × Multimorbidity (ref. no)										
YES		0.01 (−0.12; 0.14)	−0.05 (−0.08; −0.03)^***^				0.03 (−0.01; 0.07)	−0.11 (−0.21; −0.01)*		−0.03 (−0.05; −0.02)^***^
Age (ref. 73 years old) squared × Physical activity (ref. no) × Multimorbidity (ref. no)										
YES		0.12 (0.01; 0.20)*	−0.02 (−0.04; −0.001)*				0.03 (−0.002; 0.06)	−0.07 (−0.14; 0.01)		−0.003 (−0.01; 0.01)
*R^2^*m	0.62	0.62	0.28	0.28	0.18	0.18	0.33	0.33	0.32	0.32
*R*^2^c	0.86	0.86	0.55	0.55	0.52	0.52	0.64	0.64	0.60	0.60

**Notes.** Model 1a tests the independent associations of multimorbidity and physical activity with the level of the health indicators, adjusting for prior confounders. Model 2a tests the interaction between multimorbidity and physical activity on the level of the health indicators. Model 1b tests whether multimorbidity and physical activity were independently associated with the trajectories of health indicators across aging. Model 2b tests whether physical activity moderated the association between morbidity and the evolution of health across aging. R^2^m (i.e., marginal variance) and R^2^c (i.e., conditional variance) estimate the effect size for fixed effects. * *p* ≤ 0.05; ^**^p ≤ 0.01; ^***^p ≤ 0.001.

***Levels*.** As hypothesized (H1), physically active (vs. inactive) individuals exhibited significant better physical, cognitive, mental, and general health indicators (Models 1a). Moreover, as hypothesized (H2), individuals with multimorbidity (vs. without multimorbidity) exhibited the reverse pattern – poorer physical, cognitive, mental, and general health indicators (Models 1a).

***Trajectories*.** Physical, cognitive, mental (well-being), and general health indicators showed significant declines that accelerated with age, as indicated by a significant and negative effect of the quadratic effect of age, whereas depressive symptoms showed an accelerated increase with age (p < 0.001; Model 1b). As hypothesized (H4), physically active (vs. inactive) individuals showed a significantly weaker decline in cognitive and general health indicators across aging – although this protective effect on well-being became less pronounced across aging as indicated by a significant and negative interaction between the quadratic effect of age and PA status (*p* = 0.047; Model 1b). Finally, physically active (vs. inactive) individuals showed accelerated cognitive decline at older ages (*p* = 0.018; Model 1b), thereby narrowing the differences in cognitive health levels across aging between physically active (vs. inactive) individuals. Regarding multimorbidity, contrary to our hypothesis (H5), results showed that, individuals with (vs. without) multimorbidity exhibited a significant weaker decrease across aging in physical, cognitive, and general health indicators. These slower rates of decline further increased across aging for muscle strength (*p* = 0.003; Model 1b) and delayed recall (*p* < 0.001; Model 1b). Finally, the increase in depressive symptoms became more pronounced across aging in individuals with (vs. without) multimorbidity (*p* < 0.001; Model 1b). In other words, except for depressive symptoms, these findings indicated that the differences in the health indicators between individuals with and without multimorbidity shrank across aging.

### 3.3 The moderating role of multimorbidity in the associations of PA with the levels and changes of the health indicators

**Fig. 1:**
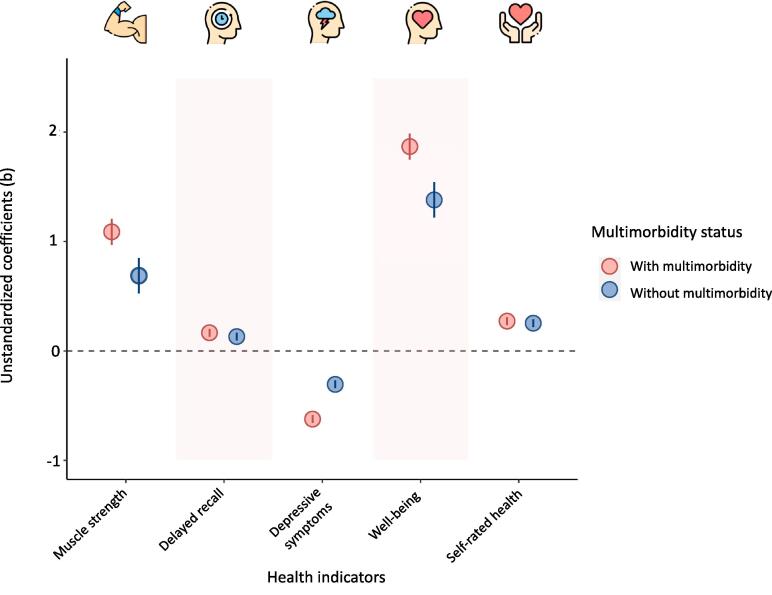
Associations of physical activity with multiple health indicators, separately for individuals with and without multimorbidity.

**Fig. 2:**
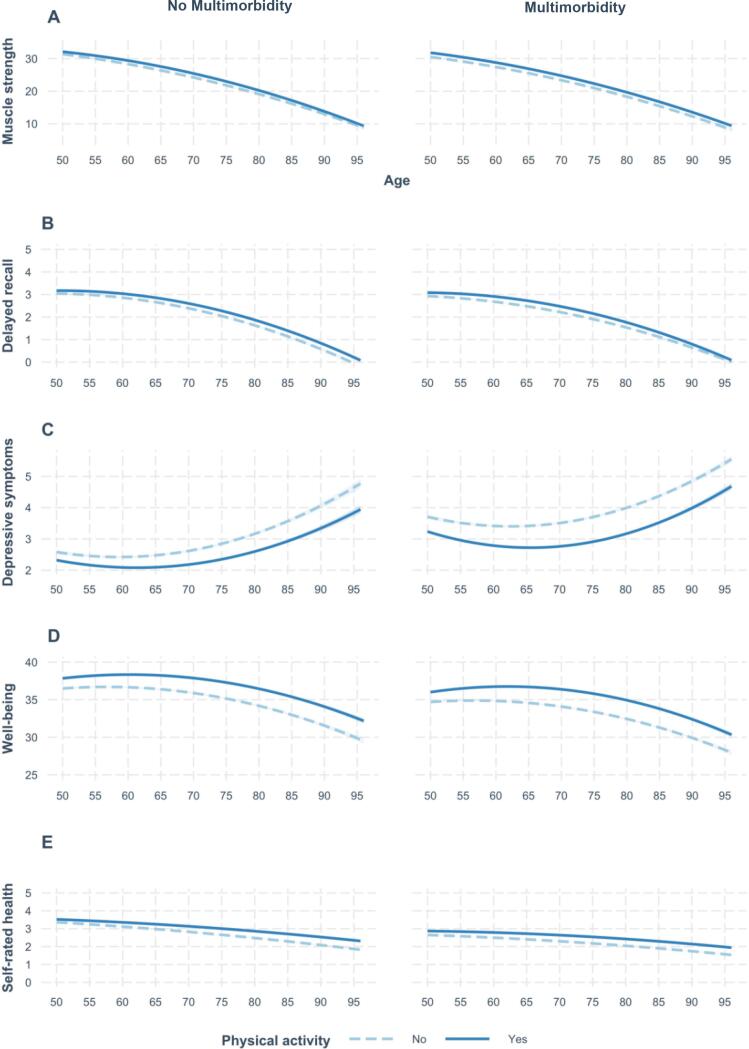
Trajectories of health indicators across aging depending on multimorbidity and physical activity status.

Table 2, Fig. 1, Fig. 2, and Supplemental Tables S2 to S6 show the interactive effects of PA and multimorbidity on levels and trajectories of the five health indicators.

***Levels.*** Interactions between PA and multimorbidity were significant for all the health indicators (*muscle strength, depressive symptoms, well-being: p* < 0.001; *delayed recall: p* = 0.003; *self-rated health: p* = 0.005; Models 2a). As hypothesized (H3), results revealed that the positive association between PA and the health indicators was significantly more pronounced in individuals with (vs. without) multimorbidity (Fig. 1).

***Trajectories.*** Results showed significant three-way interactions between PA, multimorbidity, and age (linear and quadratic) for all the health indicators except for depressive symptoms (Fig. 2). This finding suggests that the association between PA and changes in health indicators across aging, although small in magnitude, was moderated by multimorbidity. When these interactions were further analyzed, and contrary to our hypothesis (H6), results revealed that the protective role of PA in reducing age-related declines in delayed recall (*p* < 0.001; Model 2b), well-being (*p* = 0.039; Model 2b) and self-rated health (*p* < 0.001; Model 2b) became less pronounced across aging in individuals with (vs. without) multimorbidity. For delayed recall, this decrease in the effect further accelerated with advancing age as evidenced by a significant interaction with quadratic age (*p* = 0.035; Model 2b). For muscle strength, we observed that the protective role of PA in reducing its decline across aging was more pronounced in individuals with (vs. without) multimorbidity, although this effect became evident in advanced age only (*p* = 0.025; Model 2b). Overall, the percentage of marginal and conditional variance explained by this last set of models ranged from 17.5% (depressive symptoms) to 62.4% (muscle strength).

## 4 Discussion

### 4.1 Main findings

This study examined whether the positive associations between PA and health were stronger in individuals with (vs. without) multimorbidity. As hypothesized (H1 and H2), we observed that PA was associated with a better physical, cognitive, mental, and general health, while multimorbidity held a negative relationship with these health indicators. Consistent with our main hypothesis (H3), the associations between PA and health indicators were amplified among individuals with multimorbidity, as indicated by a significant interaction between multimorbidity and PA. Contrary to our hypotheses, the stronger protective role of PA on health in individuals with (vs. without) multimorbidity weakened across aging, except for depressive symptoms (no significant effect) and muscle strength (reverse pattern). Although, the effects on health indicators trajectories were of a very small effect size (Fig. 2).

### 4.2 Comparisons with other studies

Our findings confirmed the association between PA and better physical, cognitive, mental, and general health (Boisgontier et al., 2020, Cheval et al., 2020, Cimarras-Otal et al., 2014). Although our results were not consistent across all health indicators (muscle strength and cognitive health) we confirmed that PA is associated with healthy aging trajectories (Cheval et al., 2021, Feeny et al., 2014, Moreno-Agostino et al., 2020).

Our results also aligned with other studies showing that individuals with (vs. without) multimorbidity exhibit poorer health (Chudasama et al., 2019, Duggal et al., 2019, Fortin et al., 2006, Loprinzi, 2016). Our findings that individuals with (vs. without) multimorbidity had less steep decline in health indicators across aging contrast with studies reporting an association between multimorbidity and poorer health trajectories (Hsu, 2015, Vetrano et al., 2018). This discrepancy may be due to the accelerated longitudinal design, in which we examined health trajectories over a longer period of time (i.e., from 50 to 96 years) compared to most other studies (Marengoni et al., 2011). Our results may have been influenced by a selection or a survivor bias – individuals with (vs. without) multimorbidity could have been less likely to remain in the study (e.g., more likely to die). Consequently, individuals with multimorbidity who maintained their participation in the survey may represent the most resistant multimorbid individuals. Besides this methodological feature, these findings can be also be explained by the modified Strachan-Sheikh model of life course health trajectories (Strachan and Sheikh, 2004, Vineis et al., 2016). This model argues that individuals who achieved high (vs. low) health levels in their early adult-life may exhibit a more rapid rate of health decline across aging (e.g., observed in cognitive health by Aartsen et al., 2019).

Finally, we found a significant interaction between multimorbidity and PA – positive associations between PA and health indicators were amplified among individuals with multimorbidity – which complemented previous findings on the incremental protective role of PA in individuals with multimorbidity, such as a stronger effect of PA on life expectancy (Chudasama et al., 2019). This is the first formal demonstration of an increased protective role of PA on multiple components of health in individuals with multimorbidity. Except for depressive symptoms (no significant effect) and maximal muscle strength (reverse pattern), the stronger protective role of PA in individuals with multimorbidity became less pronounced with age for the other health indicators (i.e., delayed recall, well-being, and self-rated health). These findings may be explained by the fact that the health gap observed between individuals with and without multimorbidity was significantly reduced at older ages, which may have decrease the ability to detect significant differences between these individuals. However, these results on the rate of change of the health indicators across aging must be interpreted with caution as most of the interactions were above *p*-values of 0.02, providing rather weak evidence in the context of a very large sample size (Cheval et al., 2022). Furthermore, the effects of multimorbidity and PA on the trajectories of health indicators over the course of aging were very small in magnitude (Fig. 2).

### 4.3 Mechanisms at work

Although not directly measured in the current study, biological mechanisms can be proposed to explain the protective role of PA for the health of individuals, especially those suffering from multimorbidity (Duggal et al., 2019). Specifically, immunosenescence is supposed to partly explain the increased systemic inflammation that develops with age – called *inflammageing* (Franceschi and Campisi, 2014) – and that leads to worsening age-related multimorbidity (Duggal et al., 2019). Indeed, inflammation has been related with increasing risk of most chronic diseases in older age, including sarcopenia, neurodegenerative disorder, and depressive symptoms (Furman et al., 2019). As previously demonstrated, PA has extensive health benefits and is a key determinant of healthy aging (Gopinath et al., 2018). Crucially, regular PA may favor the maintenance of muscle mass and strength and, in turn, the reduction of the systemic inflammation in older age (Turner, 2016). In other words, by reducing the risk of sarcopenia (i.e., loss of muscle mass and function), PA may reduce immunosenescence (Duggal et al., 2019), thereby reducing the risk of developing age-related multimorbidity (Dalle et al., 2017, Gleeson et al., 2011). The observation that the protective role of PA was particularly pronounced in individuals with multimorbidity, in which a systemic inflammation is a key feature (Friedman and Ryff, 2012), is consistent with this hypothesis. Future studies assessing biological indicators of the immune system function (e.g., myokines and other cytokines) following a PA intervention in individuals with multimorbidity remains needed to provide direct evidence.

### 4.4 Weaknesses and strengths

This study has several limitations. First, although repeatedly used in previous studies (Boisgontier et al., 2020, Chalabaev et al., 2022, Cheval et al., 2018b, Maltagliati et al., 2021), PA was self-reported. This measure is more prone to bias than device-based measures of PA (Dyrstad et al., 2014). Future studies using a device-based measure should be conducted to confirm these findings. Similarly, chronic conditions were self-reported, which is likely biased in comparison with administrative data (Gruneir et al., 2020). Moreover, physical health was reflected by grip strength only. Additional measures, such as walking speed tests, could provide a broader view of physical health. Furthermore, we focused on the occurrence of multimorbidity (i.e., having two or more chronic conditions), but we disregarded the patterns of multimorbidity (i.e., the differences in the co-existing chronic conditions) (Lu et al., 2021). Because the different patterns of multimorbidity have been shown to exert a specific influence on health (Kim et al., 2020), future studies may also investigate multimorbidity patterns and their link to PA and health. Finally, the correlational nature of our data cannot exclude reverse causation, thereby preventing from inferring causal relationships between multimorbidity, PA, and health. It should be noted that the observed associations are likely to be bidirectional. Low PA has been shown to increase the risks of multimorbidity and increase odds of poor health, but in turn multimorbidity and poorer health may decrease engagement in PA. In other words, a vicious circle between multimorbidity, physical inactivity and poor health is likely elicited (Arias-de la Torre et al., 2021).

Despite these limitations, our large-scale longitudinal study has several strengths. The investigation of multiple health indicators (physical, cognitive, mental, and general) provided a broader view of the protective role of PA on individuals’ health. Likewise, physical and cognitive health were measured using validated tests, and mental and general health were assessed using validated scales. Furthermore, the large sample size of community-dwelling adults aged 50 and over in 28 European countries and Israel allowed for precise results with narrower confidence intervals, relative to smaller sample size studies. Finally, the longitudinal nature of the data allowed not only to investigate the relations between PA and multimorbidity on the level of health in adults aged 50 to 96 years, but also on the evolution of health across aging.

## 5 Conclusion

In conclusion, our large-scale longitudinal study of European adults aged 50 years and older suggests that the protective role of PA on multiple health indicators is particularly pronounced in individuals with (vs. without) multimorbidity, but this effect declines with advancing age. Our findings underscore the need to continue to promote PA at the population level and emphasize the need to further increase PA in people with multimorbidity.

## CRediT authorship contribution statement

**Layan Fessler:** Conceptualization, Formal analysis, Writing – original draft. **Silvio Maltagliati:** Formal analysis, Writing – review & editing. **Stefan Sieber:** Formal analysis, Writing – review & editing. **Stéphane Cullati:** Writing – review & editing. **Elena Tessitore:** Writing – review & editing. **Cecilia Craviari:** Writing – review & editing. **Christophe Luthy:** Writing – review & editing. **Eliana Hanna:** Writing – review & editing. **Philippe Meyer:** Writing – review & editing. **Dan Orsholits:** Formal analysis, Writing – review & editing. **Philippe Sarrazin:** Conceptualization, Writing – review & editing. **Boris Cheval:** Conceptualization, Supervision, Writing – original draft.

## Declaration of Competing Interest

The authors declare that they have no known competing financial interests or personal relationships that could have appeared to influence the work reported in this paper.

## Data Availability

The data are available on the Survey of Health, Ageing and Retirement in Europe (SHARE) website.
