# Supplementary material for: Physical activity matters for everyone’s health, but individuals with multimorbidity benefit more
Source: Prev Med Rep. 2023 Jun 2;34:102265. doi: 10.1016/j.pmedr.2023.102265 (PMC10240419; doi:10.1016/j.pmedr.2023.102265)
Supplement: Supplementary data 1 [file mmc1.docx]

**Supplemental materials**

**Supplemental Materials 1. Details on the measures of the health indicators.**

**Supplemental Materials 2. Descriptive statistics.**

**Supplemental Materials 3. Results of the mixed-effects models for each health indicators.**

**Supplemental Materials 1. Details on the measures of the health indicators.**

**Measures**

**Outcomes: Physical, cognitive, mental, and general health indicators.**

*Physical health* was assessed using maximal grip strength. Grip strength was measured twice for both hands using a handheld dynamometer (Smedley, S Dynamometer, TTM, Tokyo, 100 kg). The mean of the maximal values obtained for each hand at each wave was used as an indicator of muscle strength (Cheval et al., 2018), with higher values indicating higher muscle strength. Grip strength is a well-established biomarker of healthy aging (Cheval et al., 2018; Dodds et al., 2014), and has been found to predict response to acute infection and the risk of all-cause mortality (Cheval et al., 2021b; Leong et al., 2015; Maltagliati et al., 2021).

*Cognitive health* was measured using the 10-word delayed recall test (Harris and Dowson, 1982). First, participants listened to a 10-word list that was read aloud by the investigator. Then, at the end of the cognitive testing session, they were asked again to recall as many of the words from the list as possible. A higher number of words recalled indicated a higher level of cognitive health.

*Mental health* was measured by two indicators: depressive symptoms and well-being. The EURO-D scale was used to assess depressive symptoms. This scale includes 12 items indicating the presence or absence of the following features: depressed mood, pessimism, wishing death, guilt, sleep, interest, irritability, appetite, fatigue, concentration, enjoyment, and tearfulness. Each item was coded 1 (symptom present) or 0 (symptom absent) to produce a score ranging from 0 to 12. A higher score indicated greater depressive symptoms. The EURO-D scale has been shown to be reliable and valid for measuring depression in later life (Castro‐Costa et al., 2008). The short version of the CASP-19 was used to capture well-being (Hyde et al., 2003). This scale contains 12 items assessing four dimensions of individual’s needs (i.e., control, autonomy, self-realization, and pleasure). These four dimensions were combined and the resulting score was treated as a continuous variable in the analysis, with a higher score reflecting greater well-being.

*General health* was assessed using self-rated health (Ware and Sherbourne, 1992). Specifically, participants rated their own health on a five-point Likert scale ranging from 1 (poor) to 5 (excellent) by answering the following question “Would you say your health is…”. Self-rated health has been found to predict morbidity and mortality include physical, cognitive and mental health indicators (DeSalvo et al., 2006).

**Exposures: multimorbidity and physical activity**

*Multimorbidity.* Chronic conditions were assessed using the following question: “Has a doctor ever told you that you had / Do you currently have any of the conditions on this card?”. The card included a list of 17 conditions: heart attack including myocardial infarction or coronary thrombosis or any other heart problem including congestive heart failure; high blood pressure or hypertension; high blood cholesterol; stroke or cerebral vascular disease; diabetes or high blood sugar; chronic lung disease such as chronic bronchitis or emphysema; cancer or malignant or benign tumor, including leukemia or lymphoma but excluding minor skin cancers; stomach or duodenal ulcer, peptic ulcer; Parkinson’s disease; cataracts; hip fracture; other fractures; Alzheimer's disease, dementia, organic brain syndrome, senility or any other serious memory impairment; other affective or emotional disorders, including anxiety, nervous or psychiatric problems; rheumatoid arthritis; osteoarthritis, or other rheumatism; no chronic condition; or others not mentioned. In this study, we excluded cataracts, hip fractures, and other fractures. Moreover, pains in the back, knees, hips and/or other joints, and self-reported obesity (i.e., Body Mass Index (BMI) ≥ 30 Kg/m^2^) were also added as chronic conditions. We defined multimorbidity as the coexistence of ≥ 2 of the measured chronic conditions (Johnston et al., 2019), with individuals being classified as either having or not having multimorbidity.

*Physical activity* was derived from the following two questions: “*How often do you engage in activities that require a low or moderate level of energy such as gardening, cleaning the car, or going for a walk?*” and “*How often do you engage in vigorous physical activity, such as sports, heavy housework, or a job that involves physical labor?*” (Cheval et al., 2020; Cheval et al., 2019; de Souto Barreto et al., 2017). Participants answered using a four-point scale: (1) more than once a week, (2) once a week, (3) one to three times a month, (4) hardly ever or never. Participants who did not answer “more than once a week” to either item were classified as physically inactive. As described in previous research (Chalabaev et al., 2022; Boris Cheval et al., 2018), this strategy reduces the potential misclassification bias that would cause physically inactive participants to be misclassified as physically active.

**Potential confounders.** Confounders were identified using directed acyclic graphs. The identified confounders were: the wave of measurement (seven waves, i.e., “when” the participant answer to the questionnaire), the sex, the birth cohort (war [between 1914 and 1918 and between 1939 and 1945], great depression [between 1929 and 1938], no war nor economic crisis [before 1913, between 1919 and 1928, and after 1945]), attrition (no dropout, dropout [participants who responded to neither wave 7 nor wave 8]), the country of residence (Austria, Belgium, Croatia, Czech Republic, Denmark, Estonia, France, Germany, Greece, Hungary, Ireland, Israel, Italy, Luxembourg, Netherlands, Poland, Portugal, Slovenia, Spain, Sweden, Switzerland), the ability to make ends meet (with great difficulty, with some difficulty, fairly easily, easily), and education (primary, secondary, tertiary; categories based on the International Standard Classification of Education, 2006).

**Supplemental Materials 2. Participants’ characteristics by multimorbidity status at baseline.**

**Table S1. Sample characteristics by multimorbidity status at baseline participants’ characteristics at baseline.**

| **N = 121,875** | **No multimorbidity**  (N = 53,997) | | **Multimorbidity**  (N =67,878) | | ***P* value** |
| --- | --- | --- | --- | --- | --- |
| **Variables** |  | |  | |  |
| **Physical activity** (*n*; *%*) |  |  |  |  |  |
| No | 12,443 | 23.04 | 22,766 | 33.54 |  |
| Yes | 41,554 | 76.96 | 45,112 | 66.46 | <.001 |
| Age at baseline (*years; SD*) | 61.60 | 9.248 | 65.915 | 9.797 | <.001 |
| **Confounders** |  |  |  |  |  |
| Sex (*n*; *%*) |  |  |  |  |  |
| Female | 2,8165 | 52.16 | 3,8967 | 57.41 |  |
| Male | 2,5832 | 47.84 | 2,8911 | 42.59 | <.001 |
| Birth cohort (*n*; *%*) |  |  |  |  |  |
| After 1945 | 35,440 | 65.63 | 32,530 | 47.92 |  |
| Between 1919 and 1928 | 2,422 | 4.49 | 5,735 | 8.45 |  |
| Between 1929 and 1938 | 7,039 | 13.04 | 15,313 | 22.56 |  |
| Between 1939 and 1945 | 9,096 | 16.85 | 14,300 | 21.07 | <.001 |
| Attrition (*n*; *%*) |  |  |  |  |  |
| No drop out | 31,146 | 57.68 | 37,274 | 54.91 |  |
| Drop out | 18,833 | 34.88 | 20,740 | 30.55 |  |
| Death | 4,018 | 7.44 | 9,864 | 14.53 | <.001 |
| Countries (*n*; *%*) |  |  |  |  |  |
| Belgium | 3,804 | 7.04 | 5,385 | 7.93 |  |
| Austria | 2,746 | 5.09 | 3,231 | 4.76 |  |
| Bulgaria | 387 | 0.72 | 480 | 0.71 |  |
| Croatia | 1,027 | 1.90 | 1,490 | 2.20 |  |
| Cyprus | 190 | 0.35 | 300 | 0.44 |  |
| Czech Republic | 2,902 | 5.37 | 5,201 | 7.66 |  |
| Denmark | 2,820 | 5.22 | 2,676 | 3.94 |  |
| Estonia | 2,517 | 4.66 | 4,924 | 7.25 |  |
| Finland | 444 | 0.82 | 668 | 0.98 |  |
| France | 3,326 | 6.16 | 4,292 | 6.32 |  |
| Germany | 3,835 | 7.10 | 4,473 | 6.59 |  |
| Greece | 3,158 | 5.85 | 3,041 | 4.48 |  |
| Hungary | 900 | 1.67 | 2,040 | 3.01 |  |
| Ireland | 455 | 0.84 | 511 | 0.75 |  |
| Israel | 1,477 | 2.74 | 2,049 | 3.02 |  |
| Italy | 3,752 | 6.95 | 4,262 | 6.28 |  |
| Latvia | 278 | 0.51 | 451 | 0.66 |  |
| Lithuania | 495 | 0.92 | 881 | 1.30 |  |
| Luxembourg | 795 | 1.47 | 1,228 | 1.81 |  |
| Malta | 337 | 0.62 | 413 | 0.61 |  |
| Netherlands | 3,454 | 6.40 | 2,589 | 3.81 |  |
| Poland | 1,421 | 2.63 | 2,642 | 3.89 |  |
| Portugal | 702 | 1.30 | 1,302 | 1.92 |  |
| Romania | 529 | 0.98 | 698 | 1.03 |  |
| Slovakia | 565 | 1.05 | 397 | 0.58 |  |
| Slovenia | 2,149 | 3.98 | 3,083 | 4.54 |  |
| Spain | 3,724 | 6.90 | 4,404 | 6.49 |  |
| Sweden | 3,257 | 6.03 | 2,985 | 4.40 |  |
| Switzerland | 2,551 | 4.72 | 1,782 | 2.63 | <.001 |
| Able to make ends meet (*n*; *%*) |  |  |  |  |  |
| Easily | 21,604 | 40.01 | 20,201 | 29.76 |  |
| Fairly easily | 16,697 | 30.92 | 20,481 | 30.17 |  |
| With some difficulties | 10,614 | 19.66 | 16,977 | 25.01 |  |
| With great difficulties | 5,082 | 9.41 | 10,219 | 15.06 | <.001 |
| Education (*n*; *%*) |  |  |  |  |  |
| Primary | 9,663 | 17.89 | 18,459 | 27.19 |  |
| Secondary | 30,420 | 56.34 | 37,720 | 55.57 |  |
| Tertiary | 13,914 | 25.77 | 11,699 | 17.24 | <.001 |
| **Outcomes** (*mean; SD*) |  |  |  |  |  |
| Physical health |  |  |  |  |  |
| Maximal muscle strength | 36.19 | 11.79 | 32.41 | 12.25 | <.001 |
| Cognitive health |  |  |  |  |  |
| Delayed recall | 2.61 | 1.42 | 2.99 | 1.30 | <.001 |
| Mental health |  |  |  |  |  |
| Depressive symptoms | 1.82 | 1.93 | 3.01 | 2.45 | <.001 |
| Well-being | 38.7 | 5.77 | 35.55 | 6.51 | <.001 |
| General health |  |  |  |  |  |
| Self-rated health | 3.39 | 1.01 | 2.45 | 0.98 | <.001 |

**Notes.** Baseline = the first measurement occasion for each participant; SD = standard deviation, *p* values were based on the analysis of variance and chi-square tests for continuous and categorical variables, respectively, testing the effect of multimorbidity status at baseline (multimorbidity vs. no multimorbidity) on these variables. Ability to make ends meet refers to pay for the things that individuals need to live when they have little money. The descriptive statistics were based on the larger sample size (i.e., 121,875 from the models testing depressive symptoms).

**Supplemental materials 3. Results of the mixed-effects models for each health indicators.**

**Table S2. Results of the mixed-effects models for maximal muscle strength**

|  | | **Minimally adjusted *level*** | | | **Minimally adjusted**  ***INTERACTION level*** | | | **Minimally adjusted *trajectory*** | | | **Minimally adjusted *INTERACTION trajectory*** | | | **Model *1a*** | | | **Model *1b*** | | | **Model 2*a*** | | | **Model 2b** | | |
| --- | --- | --- | --- | --- | --- | --- | --- | --- | --- | --- | --- | --- | --- | --- | --- | --- | --- | --- | --- | --- | --- | --- | --- | --- | --- |
| **Variables** | Coefficient (95% CI) | | *P* value | Coefficient (95% CI) | | *P* value | Coefficient (95% CI) | | *P* value | Coefficient (95% CI) | | *P* value | Coefficient (95% CI) | | *P* value | Coefficient (95% CI) | | *P* value | Coefficient (95% CI) | | *P* value | Coefficient (95% CI) | | | *P* value |
| **Intercept** | 22.718 (22.609; 22.827) | | <0.001 | 22.840 (22.720; 22.959) | | <0.001 | 22.705 (22.587; 22.822) | | <0.001 | 22.783 (22.646; 22.920) | | <0.001 | 24.611 (24.315; 24.907) | | <0.001 | 24.734 (24.434; 25.034) | | <0.001 | 24.588 (24.289; 24.887) | | <0.001 | 24.667 (24.360; 24.974) | | <0.001 | |
| **Sex (ref. Male)** | 16.055 (15.975; 16.135) | | <0.001 | 16.052 (15.972; 16.132) | | <0.001 | 16.050 (15.970; 16.130) | | <0.001 | 16.049 (15.970; 16.129) | | <0.001 | 16.073 (15.997; 16.149) | | <0.001 | 16.070 (15.994; 16.147) | | <0.001 | 16.071 (15.994; 16.147) | | <0.001 | 16.070 (15.994; 16.146) | | <0.001 | |
| **Wave** | 0.039 (0.027; 0.051) | | <0.001 | 0.039 (0.027; 0.051) | | <0.001 | 0.038 (0.026; 0.050) | | <0.001 | 0.038 (0.026; 0.050) | | <0.001 | -0.072 (-0.095; -0.050) | | <0.001 | -0.072 (-0.095; -0.050) | | <0.001 | -0.072 (-0.095; -0.050) | | <0.001 | -0.072 (-0.095; -0.050) | | <0.001 | |
| **Country (ref. Belgium)** |  | |  |  | |  |  | |  |  | |  |  | |  |  | |  |  | |  |  | |  | |
| Austria |  | |  |  | |  |  | |  |  | |  | 0.532 (0.315; 0.748) | | <0.001 | 0.530 (0.313; 0.746) | | <0.001 | 0.531 (0.314; 0.747) | | <0.001 | 0.528 (0.312; 0.745) | | <0.001 | |
| Bulgaria |  | |  |  | |  |  | |  |  | |  | -4.662 (-5.173; -4.151) | | <0.001 | -4.668 (-5.180; -4.157) | | <0.001 | -4.656 (-5.167; -4.144) | | <0.001 | -4.666 (-5.178; -4.155) | | <0.001 | |
| Croatia |  | |  |  | |  |  | |  |  | |  | 0.361 (0.047; 0.675) | | 0.024 | 0.358 (0.044; 0.672) | | 0.025 | 0.363 (0.049; 0.677) | | 0.023 | 0.357 (0.044; 0.671) | | 0.026 | |
| Cyprus |  | |  |  | |  |  | |  |  | |  | -4.786 (-5.448; -4.124) | | <0.001 | -4.795 (-5.457; -4.133) | | <0.001 | -4.778 (-5.440; -4.116) | | <0.001 | -4.789 (-5.452; -4.127) | | <0.001 | |
| Czech Republic |  | |  |  | |  |  | |  |  | |  | 0.824 (0.624; 1.023) | | <0.001 | 0.823 (0.623; 1.022) | | <0.001 | 0.826 (0.627; 1.026) | | <0.001 | 0.824 (0.624; 1.023) | | <0.001 | |
| Denmark |  | |  |  | |  |  | |  |  | |  | 1.130 (0.910; 1.350) | | <0.001 | 1.132 (0.911; 1.352) | | <0.001 | 1.131 (0.911; 1.352) | | <0.001 | 1.132 (0.912; 1.352) | | <0.001 | |
| Estonia |  | |  |  | |  |  | |  |  | |  | 0.961 (0.755; 1.167) | | <0.001 | 0.961 (0.755; 1.167) | | <0.001 | 0.962 (0.756; 1.168) | | <0.001 | 0.960 (0.754; 1.166) | | <0.001 | |
| Finland |  | |  |  | |  |  | |  |  | |  | 0.720 (0.275; 1.164) | | 0.002 | 0.719 (0.275; 1.164) | | 0.002 | 0.723 (0.279; 1.167) | | 0.001 | 0.721 (0.277; 1.166) | | 0.001 | |
| France |  | |  |  | |  |  | |  |  | |  | -0.886 (-1.088; -0.684) | | <0.001 | -0.888 (-1.090; -0.686) | | <0.001 | -0.887 (-1.089; -0.685) | | <0.001 | -0.890 (-1.092; -0.688) | | <0.001 | |
| Germany |  | |  |  | |  |  | |  |  | |  | 0.751 (0.551; 0.950) | | <0.001 | 0.750 (0.550; 0.949) | | <0.001 | 0.751 (0.552; 0.951) | | <0.001 | 0.749 (0.550; 0.949) | | <0.001 | |
| Greece |  | |  |  | |  |  | |  |  | |  | -0.715 (-0.938; -0.492) | | <0.001 | -0.719 (-0.942; -0.496) | | <0.001 | -0.714 (-0.937; -0.491) | | <0.001 | -0.722 (-0.945; -0.499) | | <0.001 | |
| Hungary |  | |  |  | |  |  | |  |  | |  | -0.829 (-1.128; -0.529) | | <0.001 | -0.828 (-1.128; -0.528) | | <0.001 | -0.825 (-1.125; -0.526) | | <0.001 | -0.826 (-1.126; -0.526) | | <0.001 | |
| Ireland |  | |  |  | |  |  | |  |  | |  | -1.292 (-1.826; -0.759) | | <0.001 | -1.293 (-1.827; -0.760) | | <0.001 | -1.294 (-1.828; -0.761) | | <0.001 | -1.298 (-1.831; -0.764) | | <0.001 | |
| Israel |  | |  |  | |  |  | |  |  | |  | -3.966 (-4.228; -3.704) | | <0.001 | -3.965 (-4.227; -3.703) | | <0.001 | -3.966 (-4.228; -3.705) | | <0.001 | -3.968 (-4.229; -3.706) | | <0.001 | |
| Italy |  | |  |  | |  |  | |  |  | |  | -1.394 (-1.600; -1.189) | | <0.001 | -1.401 (-1.607; -1.196) | | <0.001 | -1.395 (-1.600; -1.189) | | <0.001 | -1.403 (-1.608; -1.197) | | <0.001 | |
| Latvia |  | |  |  | |  |  | |  |  | |  | 1.907 (1.344; 2.471) | | <0.001 | 1.905 (1.341; 2.469) | | <0.001 | 1.911 (1.347; 2.474) | | <0.001 | 1.907 (1.344; 2.471) | | <0.001 | |
| Lithuania |  | |  |  | |  |  | |  |  | |  | 0.961 (0.549; 1.373) | | <0.001 | 0.961 (0.549; 1.373) | | <0.001 | 0.963 (0.552; 1.375) | | <0.001 | 0.961 (0.550; 1.373) | | <0.001 | |
| Luxembourg |  | |  |  | |  |  | |  |  | |  | -0.830 (-1.159; -0.501) | | <0.001 | -0.830 (-1.159; -0.501) | | <0.001 | -0.827 (-1.156; -0.498) | | <0.001 | -0.828 (-1.157; -0.499) | | <0.001 | |
| Malta |  | |  |  | |  |  | |  |  | |  | -4.154 (-4.699; -3.608) | | <0.001 | -4.161 (-4.707; -3.616) | | <0.001 | -4.146 (-4.691; -3.600) | | <0.001 | -4.153 (-4.698; -3.608) | | <0.001 | |
| Netherlands |  | |  |  | |  |  | |  |  | |  | 0.599 (0.380; 0.818) | | <0.001 | 0.602 (0.383; 0.820) | | <0.001 | 0.599 (0.380; 0.817) | | <0.001 | 0.600 (0.381; 0.819) | | <0.001 | |
| Poland |  | |  |  | |  |  | |  |  | |  | -0.290 (-0.544; -0.036) | | 0.025 | -0.290 (-0.544; -0.036) | | 0.025 | -0.287 (-0.541; -0.033) | | 0.027 | -0.290 (-0.544; -0.036) | | 0.025 | |
| Portugal |  | |  |  | |  |  | |  |  | |  | -3.338 (-3.675; -3.001) | | <0.001 | -3.341 (-3.678; -3.004) | | <0.001 | -3.337 (-3.674; -3.000) | | <0.001 | -3.341 (-3.678; -3.004) | | <0.001 | |
| Romania |  | |  |  | |  |  | |  |  | |  | -1.799 (-2.240; -1.358) | | <0.001 | -1.802 (-2.242; -1.361) | | <0.001 | -1.793 (-2.234; -1.352) | | <0.001 | -1.798 (-2.239; -1.357) | | <0.001 | |
| Slovakia |  | |  |  | |  |  | |  |  | |  | -6.188 (-6.693; -5.682) | | <0.001 | -6.202 (-6.708; -5.696) | | <0.001 | -6.186 (-6.692; -5.680) | | <0.001 | -6.204 (-6.710; -5.698) | | <0.001 | |
| Slovenia |  | |  |  | |  |  | |  |  | |  | 0.776 (0.547; 1.006) | | <0.001 | 0.775 (0.545; 1.004) | | <0.001 | 0.777 (0.547; 1.006) | | <0.001 | 0.773 (0.544; 1.003) | | <0.001 | |
| Spain |  | |  |  | |  |  | |  |  | |  | -4.013 (-4.216; -3.811) | | <0.001 | -4.016 (-4.218; -3.813) | | <0.001 | -4.014 (-4.217; -3.812) | | <0.001 | -4.018 (-4.221; -3.815) | | <0.001 | |
| Sweden |  | |  |  | |  |  | |  |  | |  | 1.048 (0.838; 1.258) | | <0.001 | 1.049 (0.839; 1.259) | | <0.001 | 1.046 (0.836; 1.256) | | <0.001 | 1.046 (0.836; 1.256) | | <0.001 | |
| Switzerland |  | |  |  | |  |  | |  |  | |  | -0.105 (-0.341; 0.130) | | 0.381 | -0.107 (-0.342; 0.129) | | 0.375 | -0.107 (-0.342; 0.129) | | 0.374 | -0.108 (-0.344; 0.127) | | 0.367 | |
| **Birth cohort (ref. born after 1945)** |  | |  |  | |  |  | |  |  | |  |  | |  |  | |  |  | |  |  | |  | |
| 1919 to 1928 |  | |  |  | |  |  | |  |  | |  | -0.126 (-0.454; 0.202) | | 0.452 | -0.127 (-0.455; 0.201) | | 0.448 | -0.125 (-0.453; 0.203) | | 0.455 | -0.130 (-0.458; 0.198) | | 0.438 | |
| 1929 to 1938 |  | |  |  | |  |  | |  |  | |  | -0.352 (-0.558; -0.145) | | 0.001 | -0.351 (-0.558; -0.145) | | 0.001 | -0.338 (-0.545; -0.131) | | 0.001 | -0.342 (-0.549; -0.135) | | 0.001 | |
| 1939 to 1945 |  | |  |  | |  |  | |  |  | |  | -0.265 (-0.405; -0.124) | | <0.001 | -0.265 (-0.405; -0.125) | | <0.001 | -0.254 (-0.395; -0.114) | | <0.001 | -0.256 (-0.396; -0.115) | | <0.001 | |
| **Attrition (ref. No drop out)** |  | |  |  | |  |  | |  |  | |  |  | |  |  | |  |  | |  |  | |  | |
| Drop out |  | |  |  | |  |  | |  |  | |  | -0.450 (-0.543; -0.358) | | <0.001 | -0.450 (-0.543; -0.358) | | <0.001 | -0.450 (-0.543; -0.358) | | <0.001 | -0.451 (-0.543; -0.358) | | <0.001 | |
| Death |  | |  |  | |  |  | |  |  | |  | -2.483 (-2.622; -2.345) | | <0.001 | -2.479 (-2.618; -2.341) | | <0.001 | -2.480 (-2.619; -2.342) | | <0.001 | -2.479 (-2.617; -2.340) | | <0.001 | |
| **Education (ref. primary)** |  | |  |  | |  |  | |  |  | |  |  | |  |  | |  |  | |  |  | |  | |
| Secondary |  | |  |  | |  |  | |  |  | |  | 0.712 (0.605; 0.819) | | <0.001 | 0.709 (0.602; 0.816) | | <0.001 | 0.710 (0.603; 0.817) | | <0.001 | 0.708 (0.601; 0.815) | | <0.001 | |
| Tertiary |  | |  |  | |  |  | |  |  | |  | 0.879 (0.750; 1.008) | | <0.001 | 0.877 (0.748; 1.006) | | <0.001 | 0.877 (0.748; 1.006) | | <0.001 | 0.874 (0.745; 1.003) | | <0.001 | |
| **Income** |  | |  |  | |  |  | |  |  | |  |  | |  |  | |  |  | |  |  | |  | |
| Fairly easily |  | |  |  | |  |  | |  |  | |  | -0.359 (-0.457; -0.262) | | <0.001 | -0.361 (-0.459; -0.263) | | <0.001 | -0.359 (-0.456; -0.261) | | <0.001 | -0.360 (-0.458; -0.263) | | <0.001 | |
| With some difficulties |  | |  |  | |  |  | |  |  | |  | -1.253 (-1.366; -1.139) | | <0.001 | -1.252 (-1.366; -1.139) | | <0.001 | -1.251 (-1.365; -1.138) | | <0.001 | -1.251 (-1.365; -1.138) | | <0.001 | |
| With great difficulties |  | |  |  | |  |  | |  |  | |  | -2.480 (-2.624; -2.336) | | <0.001 | -2.476 (-2.620; -2.332) | | <0.001 | -2.480 (-2.624; -2.336) | | <0.001 | -2.476 (-2.620; -2.332) | | <0.001 | |
| **Level** |  | |  |  | |  |  | |  |  | |  |  | |  |  | |  |  | |  |  | |  | |
| Physical activity (ref. no) |  | |  |  | |  |  | |  |  | |  |  | |  |  | |  |  | |  |  | |  | |
| YES | 1.250 (1.198; 1.302) | | <0.001 | 1.100 (1.020; 1.180) | | <0.001 | 1.324 (1.255; 1.393) | | <0.001 | 1.220 (1.107; 1.333) | | <0.001 | 1.019 (0.967; 1.070) | | <0.001 | 0.868 (0.789; 0.948) | | <0.001 | 1.068 (1.000; 1.137) | | <0.001 | 0.972 (0.860; 1.084) | | <0.001 | |
| Multimorbidity (ref.no) |  | |  |  | |  |  | |  |  | |  |  | |  |  | |  |  | |  |  | |  | |
| YES | -0.667 (-0.717; -0.617) | | <0.001 | -0.851 (-0.941; -0.761) | | <0.001 | -0.731 (-0.798; -0.664) | | <0.001 | -0.833 (-0.956; -0.710) | | <0.001 | -0.557 (-0.607; -0.507) | | <0.001 | -0.741 (-0.830; -0.652) | | <0.001 | -0.598 (-0.664; -0.532) | | <0.001 | -0.690 (-0.812; -0.568) | | <0.001 | |
| Physical activity (ref. no) x Multimorbidity (ref. no) |  | |  |  | |  |  | |  |  | |  |  | |  |  | |  |  | |  |  | |  | |
| YES |  | |  | 0.245 (0.146; 0.345) | | <0.001 |  | |  | 0.142 (0.004; 0.280) | | 0.044 |  | |  | 0.245 (0.147; 0.344) | | <0.001 |  | |  | 0.128 (-0.008; 0.265) | | 0.066 | |
| **Rate of change** |  | |  |  | |  |  | |  |  | |  |  | |  |  | |  |  | |  |  | |  | |
| Age (ref. 73 years old) | -4.892 (-4.941; -4.843) | | <0.001 | -4.889 (-4.938; -4.839) | | <0.001 | -4.917 (-4.993; -4.841) | | <0.001 | -4.909 (-5.001; -4.816) | | <0.001 | -4.456 (-4.563; -4.349) | | <0.001 | -4.453 (-4.560; -4.346) | | <0.001 | -4.506 (-4.626; -4.385) | | <0.001 | -4.504 (-4.635; -4.372) | | <0.001 | |
| Age (ref. 73 years old) squared | -0.549 (-0.576; -0.522) | | <0.001 | -0.548 (-0.575; -0.522) | | <0.001 | -0.539 (-0.588; -0.489) | | <0.001 | -0.513 (-0.576; -0.449) | | <0.001 | -0.519 (-0.548; -0.490) | | <0.001 | -0.518 (-0.547; -0.489) | | <0.001 | -0.525 (-0.575; -0.475) | | <0.001 | -0.493 (-0.557; -0.430) | | <0.001 | |
| Age (ref. 73 years old) x Physical activity (ref. no) |  | |  |  | |  |  | |  |  | |  |  | |  |  | |  |  | |  |  | |  | |
| YES |  | |  |  | |  | -0.012 (-0.076; 0.052) | | 0.715 | -0.043 (-0.147; 0.062) | | 0.422 |  | |  |  | |  | -0.001 (-0.065; 0.063) | | 0.968 | -0.022 (-0.126; 0.082) | | 0.675 | |
| Age (ref. 73 years old) squared x Physical activity (ref. no) |  | |  |  | |  |  | |  |  | |  |  | |  |  | |  |  | |  |  | |  | |
| YES |  | |  |  | |  | -0.067 (-0.114; -0.020) | | 0.005 | -0.109 (-0.180; -0.037) | | 0.003 |  | |  |  | |  | -0.042 (-0.088; 0.005) | | 0.079 | -0.089 (-0.160; -0.018) | | 0.014 | |
| Age (ref. 73 years old) x Multimorbidity (ref. no) |  | |  |  | |  |  | |  |  | |  |  | |  |  | |  |  | |  |  | |  | |
| YES |  | |  |  | |  | 0.046 (-0.021; 0.114) | | 0.179 | 0.056 (- 0.048; 0.159) | | 0.292 |  | |  |  | |  | 0.073 (0.005; 0.140) | | 0.034 | 0.094 (-0.008; 0.197) | | 0.071 | |
| Age (ref. 73 years old) squared x Multimorbidity (ref. no) |  | |  |  | |  |  | |  |  | |  |  | |  |  | |  |  | |  |  | |  | |
| YES |  | |  |  | |  | 0.074 (0.029; 0.119) | | 0.001 | 0.016 (-0.062; 0.095) | | 0.683 |  | |  |  | |  | 0.069 (0.024; 0.114) | | 0.003 | -0.000 (-0.078; 0.077) | | 0.991 | |
| Age (ref. 73 years old) x Physical activity (ref. no) x Multimorbidity (ref. no) |  | |  |  | |  |  | |  |  | |  |  | |  |  | |  |  | |  |  | |  | |
| YES |  | |  |  | |  |  | |  | 0.026 (-0.102; 0.154) | | 0.691 |  | |  |  | |  |  | |  | 0.009 (-0.118; 0.136) | | 0.892 | |
| Age (ref. 73 years old) squared x Physical activity (ref. no) x Multimorbidity (ref. no) |  | |  |  | |  |  | |  |  | |  |  | |  |  | |  |  | |  |  | |  | |
| YES |  | |  |  | |  |  | |  | 0.091 (-0.002; 0.183) | | 0.055 |  | |  |  | |  |  | |  | 0.105 (0.013; 0.197) | | 0.025 | |
| *R^2^*m | 0.622 | |  | 0.590 | |  | 0.590 | |  | 0.590 | |  | 0.624 | |  | 0.624 | |  | 0.623 | |  | 0.624 | |  | |
| *R*^2^c | 0.860 | |  | 0.862 | |  | 0.862 | |  | 0.862 | |  | 0.862 | |  | 0.862 | |  | 0.862 | |  | 0.862 | |  | |

**Note.** Minimally adjusted *level* = minimally adjusted model (i.e., age, sex, and wave) testing the independent association of multimorbidity and physical activity on the level of health indicators; Minimally adjusted *INTERACTION level* = minimally adjusted model testing the interactive association of multimorbidity and physical activity on the level of health indicators; Minimally adjusted *trajectory* = minimally adjusted model testing the independent association of multimorbidity and physical activity on the level and rate of change of the health indicators across aging; Minimally adjusted *INTERACTION trajectory* = minimally adjusted model testing the interactive association of multimorbidity and physical activity on the level and rate of change of the health indicators across aging; Models 1a, 1b, 2a, and 2b are the full results for the models tested in the main analysis.

**Table S3. Results of the mixed-effects models for delayed recall**

|  | **Minimally adjusted *level*** | | **Minimally adjusted**  ***INTERACTION level*** | | **Minimally adjusted *trajectory*** | | **Minimally adjusted *INTERACTION trajectory*** | | **Model *1a*** | | **Model *1b*** | | **Model 2*a*** | | **Model 2b** | |
| --- | --- | --- | --- | --- | --- | --- | --- | --- | --- | --- | --- | --- | --- | --- | --- | --- |
| **Variables** | Coefficient (95% CI) | *P* value | Coefficient (95% CI) | *P* value | Coefficient (95% CI) | *P* value | Coefficient (95% CI) | *P* value | Coefficient (95% CI) | *P* value | Coefficient (95% CI) | *P* value | Coefficient (95% CI) | *P* value | Coefficient (95% CI) | *P* value |
| **Intercept** | 2.190 (2.170; 2.210) | <0.001 | 2.204 (2.181; 2.226) | <0.001 | 2.175 (2.153; 2.197) | <0.001 | 2.192 (2.165; 2.218) | <0.001 | -0.130 (-0.136; -0.124) | <0.001 | -0.130 (-0.135; -0.124) | <0.001 | -0.132 (-0.142; -0.122) | <0.001 | -0.141 (-0.154; -0.129) | <0.001 |
| **Sex (ref. Male)** | -0.135 (-0.140; -0.130) | <0.001 | -0.135 (-0.140; -0.130) | <0.001 | -0.135 (-0.145; -0.125) | <0.001 | -0.145 (-0.157; -0.132) | <0.001 | -0.190 (-0.202; -0.179) | <0.001 | -0.190 (-0.202; -0.179) | <0.001 | -0.191 (-0.202; -0.179) | <0.001 | -0.191 (-0.202; -0.179) | <0.001 |
| **Wave** |  |  |  |  |  |  |  |  |  | <0.001 | 0.056 (0.052; 0.059) | <0.001 | 0.056 (0.052; 0.059) | <0.001 | 0.056 (0.052; 0.059) | <0.001 |
| **Country (ref. Belgium)** |  |  |  |  |  |  |  |  |  |  |  |  |  |  |  |  |
| Austria |  |  |  |  |  |  |  |  | 0.174 (0.142; 0.206) | <0.001 | 0.174 (0.142; 0.206) | <0.001 | 0.174 (0.142; 0.206) | <0.001 | 0.174 (0.142; 0.206) | <0.001 |
| Bulgaria |  |  |  |  |  |  |  |  | -0.055 (-0.149; 0.039) | 0.255 | -0.055 (-0.149; 0.039) | 0.249 | -0.053 (-0.147; 0.041) | 0.27 | -0.054 (-0.148; 0.040) | 0.256 |
| Croatia |  |  |  |  |  |  |  |  | -0.267 (-0.317; -0.216) | <0.001 | -0.267 (-0.317; -0.217) | <0.001 | -0.265 (-0.316; -0.215) | <0.001 | -0.266 (-0.317; -0.216) | <0.001 |
| Cyprus |  |  |  |  |  |  |  |  | -0.447 (-0.564; -0.330) | <0.001 | -0.448 (-0.565; -0.331) | <0.001 | -0.444 (-0.561; -0.327) | <0.001 | -0.444 (-0.562; -0.327) | <0.001 |
| Czech Republic |  |  |  |  |  |  |  |  | -0.030 (-0.059; 0.000) | 0.054 | -0.030 (-0.060; 0.000) | 0.052 | -0.029 (-0.059; 0.001) | 0.059 | -0.029 (-0.059; 0.001) | 0.056 |
| Denmark |  |  |  |  |  |  |  |  | 0.152 (0.120; 0.184) | <0.001 | 0.152 (0.120; 0.184) | <0.001 | 0.153 (0.121; 0.185) | <0.001 | 0.153 (0.121; 0.186) | <0.001 |
| Estonia |  |  |  |  |  |  |  |  | -0.108 (-0.139; -0.078) | <0.001 | -0.108 (-0.139; -0.078) | <0.001 | -0.108 (-0.138; -0.077) | <0.001 | -0.108 (-0.139; -0.077) | <0.001 |
| Finland |  |  |  |  |  |  |  |  | -0.114 (-0.194; -0.033) | 0.006 | -0.114 (-0.194; -0.033) | 0.006 | -0.113 (-0.193; -0.032) | 0.006 | -0.113 (-0.194; -0.032) | 0.006 |
| France |  |  |  |  |  |  |  |  | 0.032 (0.002; 0.062) | 0.034 | 0.032 (0.002; 0.061) | 0.036 | 0.031 (0.002; 0.061) | 0.038 | 0.031 (0.001; 0.061) | 0.04 |
| Germany |  |  |  |  |  |  |  |  | 0.026 (-0.004; 0.055) | 0.093 | 0.025 (-0.004; 0.055) | 0.093 | 0.026 (-0.004; 0.056) | 0.085 | 0.026 (-0.004; 0.056) | 0.087 |
| Greece |  |  |  |  |  |  |  |  | 0.097 (0.063; 0.130) | <0.001 | 0.096 (0.063; 0.130) | <0.001 | 0.097 (0.064; 0.131) | <0.001 | 0.096 (0.062; 0.130) | <0.001 |
| Hungary |  |  |  |  |  |  |  |  | -0.053 (-0.102; -0.004) | 0.033 | -0.053 (-0.102; -0.004) | 0.033 | -0.052 (-0.101; -0.003) | 0.038 | -0.052 (-0.101; -0.003) | 0.037 |
| Ireland |  |  |  |  |  |  |  |  | 0.176 (0.093; 0.259) | <0.001 | 0.176 (0.093; 0.259) | <0.001 | 0.176 (0.093; 0.258) | <0.001 | 0.175 (0.093; 0.258) | <0.001 |
| Israel |  |  |  |  |  |  |  |  | -0.047 (-0.086; -0.008) | 0.019 | -0.047 (-0.086; -0.008) | 0.019 | -0.047 (-0.086; -0.008) | 0.018 | -0.047 (-0.086; -0.008) | 0.018 |
| Italy |  |  |  |  |  |  |  |  | -0.172 (-0.202; -0.141) | <0.001 | -0.172 (-0.203; -0.142) | <0.001 | -0.172 (-0.203; -0.141) | <0.001 | -0.173 (-0.204; -0.143) | <0.001 |
| Latvia |  |  |  |  |  |  |  |  | -0.400 (-0.498; -0.303) | <0.001 | -0.400 (-0.498; -0.303) | <0.001 | -0.399 (-0.497; -0.302) | <0.001 | -0.400 (-0.497; -0.302) | <0.001 |
| Lithuania |  |  |  |  |  |  |  |  | -0.731 (-0.802; -0.659) | <0.001 | -0.731 (-0.803; -0.660) | <0.001 | -0.730 (-0.802; -0.659) | <0.001 | -0.731 (-0.802; -0.660) | <0.001 |
| Luxembourg |  |  |  |  |  |  |  |  | 0.122 (0.071; 0.172) | <0.001 | 0.122 (0.071; 0.172) | <0.001 | 0.123 (0.073; 0.173) | <0.001 | 0.123 (0.073; 0.173) | <0.001 |
| Malta |  |  |  |  |  |  |  |  | ; 0.489 (-0.587; -0.391) | <0.001 | -0.490 (-0.588; -0.392) | <0.001 | -0.486 (-0.584; -0.388) | <0.001 | -0.488 (-0.586; -0.390) | <0.001 |
| Netherlands |  |  |  |  |  |  |  |  | 0.102 (0.069; 0.135) | <0.001 | 0.102 (0.069; 0.135) | <0.001 | 0.102 (0.069; 0.135) | <0.001 | 0.102 (0.070; 0.135) | <0.001 |
| Poland |  |  |  |  |  |  |  |  | -0.483 (-0.522; -0.444) | <0.001 | -0.483 (-0.522; -0.445) | <0.001 | -0.482 (-0.521; -0.443) | <0.001 | -0.482 (-0.521; -0.444) | <0.001 |
| Portugal |  |  |  |  |  |  |  |  | -0.176 (-0.229; -0.123) | <0.001 | -0.177 (-0.230; -0.124) | <0.001 | -0.176 (-0.229; -0.123) | <0.001 | -0.177 (-0.230; -0.124) | <0.001 |
| Romania |  |  |  |  |  |  |  |  | -0.491 (-0.566; -0.415) | <0.001 | -0.491 (-0.566; -0.415) | <0.001 | -0.489 (-0.564; -0.413) | <0.001 | -0.489 (-0.564; -0.413) | <0.001 |
| Slovakia |  |  |  |  |  |  |  |  | -0.240 (-0.323; -0.157) | <0.001 | -0.242 (-0.325; -0.158) | <0.001 | -0.240 (-0.323; -0.157) | <0.001 | -0.243 (-0.326; -0.160) | <0.001 |
| Slovenia |  |  |  |  |  |  |  |  | -0.375 (-0.409; -0.340) | <0.001 | -0.375 (-0.410; -0.340) | <0.001 | -0.374 (-0.409; -0.339) | <0.001 | -0.374 (-0.409; -0.339) | <0.001 |
| Spain |  |  |  |  |  |  |  |  | -0.382 (-0.412; -0.351) | <0.001 | -0.382 (-0.412; -0.352) | <0.001 | -0.382 (-0.412; -0.352) | <0.001 | -0.382 (-0.412; -0.352) | <0.001 |
| Sweden |  |  |  |  |  |  |  |  | 0.233 (0.201; 0.265) | <0.001 | 0.233 (0.201; 0.265) | <0.001 | 0.233 (0.201; 0.264) | <0.001 | 0.233 (0.201; 0.265) | <0.001 |
| Switzerland |  |  |  |  |  |  |  |  | 0.290 (0.255; 0.325) | <0.001 | 0.290 (0.255; 0.325) | <0.001 | 0.290 (0.255; 0.324) | <0.001 | 0.289 (0.255; 0.324) | <0.001 |
| **Birth cohort (ref. born after 1945)** |  |  |  |  |  |  |  |  |  |  |  |  |  |  |  |  |
| 1919 to 1928 |  |  |  |  |  |  |  |  | 0.091 (0.036; 0.145) | 0.001 | 0.091 (0.036; 0.145) | 0.001 | 0.091 (0.037; 0.146) | 0.001 | 0.091 (0.037; 0.145) | 0.001 |
| 1929 to 1938 |  |  |  |  |  |  |  |  | -0.024 (-0.057-0.009) | 0.152 | -0.024 (-0.057-0.009) | 0.153 | -0.020 (-0.053-0.013) | 0.24 | -0.020 (-0.053-0.013) | 0.232 |
| 1939 to 1945 |  |  |  |  |  |  |  |  | -0.015 (-0.037-0.007) | 0.17 | -0.015 (-0.037-0.006) | 0.168 | -0.013 (-0.034-0.009) | 0.262 | -0.013 (-0.035-0.009) | 0.25 |
| **Attrition (ref. No drop out)** |  |  |  |  |  |  |  |  |  |  |  |  |  |  |  |  |
| Drop out |  |  |  |  |  |  |  |  | -0.139 (-0.153; -0.124) | <0.001 | -0.139 (-0.153; -0.124) | <0.001 | -0.138 (-0.153; -0.124) | <0.001 | -0.138 (-0.153; -0.124) | <0.001 |
| Death |  |  |  |  |  |  |  |  | -0.323 (-0.345; -0.300) | <0.001 | -0.322 (-0.345; -0.300) | <0.001 | -0.321 (-0.344; -0.299) | <0.001 | -0.321 (-0.343; -0.299) | <0.001 |
| **Education (ref. primary)** |  |  |  |  |  |  |  |  |  |  |  |  |  |  |  |  |
| Secondary |  |  |  |  |  |  |  |  | 0.462 (0.445; 0.478) | <0.001 | 0.461 (0.445; 0.478) | <0.001 | 0.461 (0.444; 0.478) | <0.001 | 0.461 (0.444; 0.477) | <0.001 |
| Tertiary |  |  |  |  |  |  |  |  | 0.783 (0.763; 0.803) | <0.001 | 0.782 (0.763; 0.802) | <0.001 | 0.782 (0.762; 0.802) | <0.001 | 0.782 (0.762; 0.802) | <0.001 |
| **Income** |  |  |  |  |  |  |  |  |  |  |  |  |  |  |  |  |
| Fairly easily |  |  |  |  |  |  |  |  | -0.048 (-0.063; -0.033) | <0.001 | -0.048 (-0.063; -0.034) | <0.001 | -0.048 (-0.063; -0.033) | <0.001 | -0.048 (-0.063; -0.034) | <0.001 |
| With some difficulties |  |  |  |  |  |  |  |  | -0.138 (-0.156; -0.121) | <0.001 | -0.138 (-0.156; -0.121) | <0.001 | -0.138 (-0.156; -0.121) | <0.001 | -0.138 (-0.156; -0.121) | <0.001 |
| With great difficulties |  |  |  |  |  |  |  |  | -0.351 (-0.373; -0.329) | <0.001 | -0.350 (-0.372; -0.329) | <0.001 | -0.351 (-0.373; -0.330) | <0.001 | -0.351 (-0.372; -0.329) | <0.001 |
| **Level** |  |  |  |  |  |  |  |  |  |  |  |  |  |  |  |  |
| Physical activity (ref. no) |  |  |  |  |  |  |  |  |  |  |  |  |  |  |  |  |
| YES | 0.216 (0.206; 0.227) | <0.001 | 0.199 (0.182; 0.216) | <0.001 | 0.242 (0.228; 0.257) | <0.001 | 0.222 (0.198; 0.246) | <0.001 | 0.145 (0.134; 0.155) | <0.001 | 0.126 (0.109; 0.142) | <0.001 | 0.164 (0.150; 0.179) | <0.001 | 0.148 (0.124; 0.171) | <0.001 |
| Multimorbidity (ref.no) |  |  |  |  |  |  |  |  |  |  |  |  |  |  |  |  |
| YES | -0.119 (-0.130; -0.109) | <0.001 | -0.140 (-0.158; -0.122) | <0.001 | -0.126 (-0.140; -0.112) | <0.001 | -0.151 (-0.176; -0.125) | <0.001 | -0.048 (-0.058; -0.038) | <0.001 | -0.071 (-0.088; -0.053) | <0.001 | -0.060 (-0.074; -0.046) | <0.001 | -0.081 (-0.106; -0.056) | <0.001 |
| Physical activity (ref. no) x Multimorbidity (ref. no) |  |  |  |  |  |  |  |  |  |  |  |  |  |  |  |  |
| YES |  |  | 0.028 (0.007; 0.049) | 0.008 |  |  | 0.031 (0.002; 0.060) | 0.039 |  |  | 0.031 (0.011; 0.051) | 0.003 |  |  | 0.026 (- 0.003; 0.054) | 0.082 |
| **Rate of change** |  |  |  |  |  |  |  |  |  |  |  |  |  |  |  |  |
| Age (ref. 73 years old) | -0.657 (-0.666; -0.648) | <0.001 | -0.657 (-0.666; -0.648) | <0.001 | -0.684 (-0.698; -0.669) | <0.001 | -0.702 (-0.719; -0.684) | <0.001 | -0.571 (-0.589; -0.553) | <0.001 | -0.570 (-0.588; -0.553) | <0.001 | -0.593 (-0.613; -0.572) | <0.001 | -0.613 (-0.636; -0.589) | <0.001 |
| Age (ref. 73 years old) squared | -0.135 (-0.140; -0.130) | <0.001 | -0.135 (-0.140; -0.130) | <0.001 | -0.135 (-0.145; -0.125) | <0.001 | -0.145 (-0.157; -0.132) | <0.001 | -0.130 (-0.136; -0.124) | <0.001 | -0.130 (-0.135; -0.124) | <0.001 | -0.132 (-0.142; -0.122) | <0.001 | -0.141 (-0.154; -0.129) | <0.001 |
| Age (ref. 73 years old) x Physical activity (ref. no) |  |  |  |  |  |  |  |  |  |  |  |  |  |  |  |  |
| YES |  |  |  |  | 0.002 (-0.011; 0.015) | 0.752 | 0.030 (0.008; 0.051) | 0.007 |  |  |  |  | 0.009 (-0.004; 0.022) | 0.177 | 0.040 (0.019; 0.061) | <0.001 |
| Age (ref. 73 years old) squared x Physical activity (ref. no) |  |  |  |  |  |  |  |  |  |  |  |  |  |  |  |  |
| YES |  |  |  |  | -0.019 (-0.028; -0.010) | <0.001 | -0.004 (-0.019; 0.010) | 0.56 |  |  |  |  | -0.011 (-0.020; -0.002) | 0.018 | 0.003 (-0.011; 0.017) | 0.647 |
| Age (ref. 73 years old) x Multimorbidity (ref. no) |  |  |  |  |  |  |  |  |  |  |  |  |  |  |  |  |
| YES |  |  |  |  | 0.039 (0.025; 0.052) | <0.001 | 0.066 (0.046; 0.086) | <0.001 |  |  |  |  | 0.024 (0.011; 0.038) | <0.001 | 0.055 (0.035; 0.075) | <0.001 |
| Age (ref. 73 years old) squared x Multimorbidity (ref. no) |  |  |  |  |  |  |  |  |  |  |  |  |  |  |  |  |
| YES |  |  |  |  | 0.024 (0.015; 0.033) | <0.001 | 0.036 (0.021; 0.051) | <0.001 |  |  |  |  | 0.021 (0.012; 0.029) | <0.001 | 0.031 (0.016; 0.046) | <0.001 |
| Age (ref. 73 years old) x Physical activity (ref. no) x Multimorbidity (ref. no) |  |  |  |  |  |  |  |  |  |  |  |  |  |  |  |  |
| YES |  |  |  |  |  |  | -0.045 (-0.071; -0.018) | 0.001 |  |  |  |  |  |  | -0.051 (-0.077; -0.025) | <0.001 |
| Age (ref. 73 years old) squared x Physical activity (ref. no) x Multimorbidity (ref. no) |  |  |  |  |  |  |  |  |  |  |  |  |  |  |  |  |
| YES |  |  |  |  |  |  | -0.021 (-0.040; -0.002) | 0.029 |  |  |  |  |  |  | -0.020 (-0.038; -0.001) | 0.035 |
| *R^2^*m | 0.182 |  | 0.182 |  | 0.182 |  | 0.182 |  | 0.279 |  | 0.279 |  | 0.279 |  | 0.279 |  |
| *R*^2^c | 0.548 |  | 0.548 |  | 0.548 |  | 0.548 |  | 0.548 |  | 0.548 |  | 0.548 |  | 0.548 |  |

**Note.** Minimally adjusted *level* = minimally adjusted model (i.e., age, sex, and wave) testing the independent association of multimorbidity and physical activity on the level of health indicators; Minimally adjusted *INTERACTION level* = minimally adjusted model testing the interactive association of multimorbidity and physical activity on the level of health indicators; Minimally adjusted *trajectory* = minimally adjusted model testing the independent association of multimorbidity and physical activity on the level and rate of change of the health indicators across aging; Minimally adjusted *INTERACTION trajectory* = minimally adjusted model testing the interactive association of multimorbidity and physical activity on the level and rate of change of the health indicators across aging; Models 1a, 1b, 2a, and 2b are the full results for the models tested in the main analysis.

**Table S4. Results of the mixed-effects models for depressive symptoms**

|  | **Minimally adjusted *level*** | | **Minimally adjusted**  ***INTERACTION level*** | | **Minimally adjusted *trajectory*** | | **Minimally adjusted *INTERACTION trajectory*** | | **Model *1a*** | | **Model *1b*** | | **Model 2*a*** | | **Model 2b** | |
| --- | --- | --- | --- | --- | --- | --- | --- | --- | --- | --- | --- | --- | --- | --- | --- | --- |
| **Variables** | Coefficient (95% CI) | *P* value | Coefficient (95% CI) | *P* value | Coefficient (95% CI) | *P* value | Coefficient (95% CI) | *P* value | Coefficient (95% CI) | *P* value | Coefficient (95% CI) | *P* value | Coefficient (95% CI) | *P* value | Coefficient (95% CI) | *P* value |
| **Intercept** | 2.799 (2.768; 2.830) | <0.001 | 2.653 (2.618; 2.688) | <0.001 | 2.902 (2.868; 2.936) | <0.001 | 2.745 (2.704; 2.785) | <0.001 | 2.724 (2.647; 2.801) | <0.001 | 2.585 (2.506; 2.663) | <0.001 | 2.783 (2.705; 2.861) | <0.001 | 2.639 (2.557; 2.720) | <0.001 |
| **Sex (ref. Male)** | -0.755 (-0.776; -0.735) | <0.001 | -0.753 (-0.774; -0.733) | <0.001 | -0.754 (-0.775; -0.734) | <0.001 | -0.753 (-0.774; -0.733) | <0.001 | -0.726 (-0.746; -0.707) | <0.001 | -0.724 (-0.744; -0.705) | <0.001 | -0.725 (-0.745; -0.705) | <0.001 | -0.724 (-0.743; -0.704) | <0.001 |
| **Wave** | -0.001 (-0.004; 0.003) | 0.729 | -0.001 (-0.004; 0.003) | 0.768 | -0.001 (-0.005; 0.003) | 0.567 | -0.001 (-0.005; 0.003) | 0.596 | 0.014 (0.008; 0.020) | <0.001 | 0.014 (0.008; 0.020) | <0.001 | 0.015 (0.009; 0.022) | <0.001 | 0.015 (0.009; 0.021) | <0.001 |
| **Country (ref. Belgium)** |  |  |  |  |  |  |  |  |  |  |  |  |  |  |  |  |
| Austria |  |  |  |  |  |  |  |  | -0.414 (-0.468; -0.359) | <0.001 | -0.412 (-0.466; -0.357) | <0.001 | -0.412 (-0.466; -0.357) | <0.001 | -0.410 (-0.465; -0.356) | <0.001 |
| Bulgaria |  |  |  |  |  |  |  |  | -0.755 (-0.898; -0.613) | <0.001 | -0.748 (-0.891; -0.606) | <0.001 | -0.752 (-0.895; -0.610) | <0.001 | -0.744 (-0.887; -0.602) | <0.001 |
| Croatia |  |  |  |  |  |  |  |  | -0.383 (-0.466; -0.301) | <0.001 | -0.379 (-0.461; -0.296) | <0.001 | -0.383 (-0.466; -0.300) | <0.001 | -0.378 (-0.461; -0.296) | <0.001 |
| Cyprus |  |  |  |  |  |  |  |  | -1.168 (-1.358; -0.978) | <0.001 | -1.156 (-1.346; -0.967) | <0.001 | -1.171 (-1.360; -0.981) | <0.001 | -1.158 (-1.347; -0.968) | <0.001 |
| Czech Republic |  |  |  |  |  |  |  |  | -0.439 (-0.490; -0.388) | <0.001 | -0.438 (-0.489; -0.387) | <0.001 | -0.435 (-0.486; -0.384) | <0.001 | -0.433 (-0.484; -0.382) | <0.001 |
| Denmark |  |  |  |  |  |  |  |  | -0.398 (-0.454; -0.342) | <0.001 | -0.401 (-0.457; -0.345) | <0.001 | -0.400 (-0.456; -0.344) | <0.001 | -0.402 (-0.457; -0.346) | <0.001 |
| Estonia |  |  |  |  |  |  |  |  | 0.203 (0.150; 0.256) | <0.001 | 0.203 (0.151; 0.256) | <0.001 | 0.203 (0.150; 0.255) | <0.001 | 0.203 (0.151; 0.256) | <0.001 |
| Finland |  |  |  |  |  |  |  |  | -0.042 (-0.169; 0.084) | 0.51 | -0.041 (-0.168; 0.085) | 0.521 | -0.038 (-0.164; 0.089) | 0.559 | -0.037 (-0.164; 0.089) | 0.561 |
| France |  |  |  |  |  |  |  |  | 0.179 (0.127; 0.230) | <0.001 | 0.182 (0.130; 0.233) | <0.001 | 0.184 (0.132; 0.235) | <0.001 | 0.186 (0.134; 0.237) | <0.001 |
| Germany |  |  |  |  |  |  |  |  | -0.143 (-0.193; -0.092) | <0.001 | -0.142 (-0.192; -0.091) | <0.001 | -0.143 (-0.194; -0.092) | <0.001 | -0.141 (-0.192; -0.091) | <0.001 |
| Greece |  |  |  |  |  |  |  |  | -1.023 (-1.080; -0.966) | <0.001 | -1.017 (-1.074; -0.961) | <0.001 | -1.014 (-1.071; -0.957) | <0.001 | -1.008 (-1.065; -0.951) | <0.001 |
| Hungary |  |  |  |  |  |  |  |  | -0.114 (-0.193; -0.034) | 0.005 | -0.113 (-0.193; -0.034) | 0.005 | -0.117 (-0.197; -0.037) | 0.004 | -0.117 (-0.196; -0.037) | 0.004 |
| Ireland |  |  |  |  |  |  |  |  | -0.558 (-0.692; -0.423) | <0.001 | -0.558 (-0.693; -0.424) | <0.001 | -0.555 (-0.690; -0.421) | <0.001 | -0.556 (-0.690; -0.421) | <0.001 |
| Israel |  |  |  |  |  |  |  |  | -0.175 (-0.241; -0.109) | <0.001 | -0.175 (-0.241; -0.109) | <0.001 | -0.169 (-0.235; -0.103) | <0.001 | -0.169 (-0.235; -0.103) | <0.001 |
| Italy |  |  |  |  |  |  |  |  | -0.184 (-0.236; -0.132) | <0.001 | -0.176 (-0.228; -0.124) | <0.001 | -0.174 (-0.225; -0.122) | <0.001 | -0.167 (-0.218; -0.115) | <0.001 |
| Latvia |  |  |  |  |  |  |  |  | -0.351 (-0.505; -0.196) | <0.001 | -0.349 (-0.504; -0.195) | <0.001 | -0.347 (-0.502; -0.193) | <0.001 | -0.345 (-0.499; -0.190) | <0.001 |
| Lithuania |  |  |  |  |  |  |  |  | 0.304 (0.188; 0.420) | <0.001 | 0.306 (0.190; 0.421) | <0.001 | 0.304 (0.188; 0.420) | <0.001 | 0.306 (0.190; 0.422) | <0.001 |
| Luxembourg |  |  |  |  |  |  |  |  | 0.059 (-0.026; 0.143) | 0.174 | 0.059 (-0.026; 0.143) | 0.173 | 0.057 (-0.027; 0.142) | 0.184 | 0.058 (-0.027; 0.142) | 0.18 |
| Malta |  |  |  |  |  |  |  |  | -0.095 (-0.247; 0.056) | 0.218 | -0.085 (-0.237; 0.066) | 0.268 | -0.093 (-0.244; 0.058) | 0.229 | -0.082 (-0.234; 0.069) | 0.286 |
| Netherlands |  |  |  |  |  |  |  |  | -0.287 (-0.343; -0.231) | <0.001 | -0.291 (-0.347; -0.235) | <0.001 | -0.289 (-0.345; -0.232) | <0.001 | -0.291 (-0.347; -0.235) | <0.001 |
| Poland |  |  |  |  |  |  |  |  | 0.273 (0.207; 0.339) | <0.001 | 0.274 (0.208; 0.339) | <0.001 | 0.274 (0.208; 0.340) | <0.001 | 0.275 (0.210; 0.341) | <0.001 |
| Portugal |  |  |  |  |  |  |  |  | 0.183 (0.095; 0.271) | <0.001 | 0.189 (0.101; 0.277) | <0.001 | 0.187 (0.099; 0.275) | <0.001 | 0.192 (0.104; 0.279) | <0.001 |
| Romania |  |  |  |  |  |  |  |  | -0.075 (-0.196; 0.046) | 0.225 | -0.072 (-0.193; 0.049) | 0.243 | -0.075 (-0.196; 0.047) | 0.228 | -0.071 (-0.193; 0.050) | 0.248 |
| Slovakia |  |  |  |  |  |  |  |  | -0.314 (-0.448; -0.180) | <0.001 | -0.301 (-0.435; -0.167) | <0.001 | -0.298 (-0.432; -0.164) | <0.001 | -0.285 (-0.419; -0.151) | <0.001 |
| Slovenia |  |  |  |  |  |  |  |  | -0.446 (-0.505; -0.386) | <0.001 | -0.443 (-0.503; -0.384) | <0.001 | -0.445 (-0.504; -0.386) | <0.001 | -0.443 (-0.502; -0.384) | <0.001 |
| Spain |  |  |  |  |  |  |  |  | -0.235 (-0.287; -0.183) | <0.001 | -0.232 (-0.284; -0.180) | <0.001 | -0.230 (-0.282; -0.178) | <0.001 | -0.227 (-0.279; -0.175) | <0.001 |
| Sweden |  |  |  |  |  |  |  |  | -0.313 (-0.367; -0.259) | <0.001 | -0.314 (-0.368; -0.260) | <0.001 | -0.310 (-0.364; -0.256) | <0.001 | -0.312 (-0.366; -0.258) | <0.001 |
| Switzerland |  |  |  |  |  |  |  |  | -0.311 (-0.371; -0.251) | <0.001 | -0.310 (-0.370; -0.250) | <0.001 | -0.307 (-0.367; -0.247) | <0.001 | -0.306 (-0.366; -0.246) | <0.001 |
| **Birth cohort (ref. born after 1945)** |  |  |  |  |  |  |  |  |  |  |  |  |  |  |  |  |
| 1919 to 1928 |  |  |  |  |  |  |  |  | -0.185 (-0.274; -0.095) | <0.001 | -0.183 (-0.272; -0.094) | <0.001 | -0.164 (-0.254; -0.075) | <0.001 | -0.162 (-0.251; -0.072) | <0.001 |
| 1929 to 1938 |  |  |  |  |  |  |  |  | -0.011 (-0.065; 0.044) | 0.701 | -0.011 (-0.065; 0.043) | 0.7 | 0.015 (-0.039; 0.070) | 0.582 | 0.015 (-0.039; 0.070) | 0.58 |
| 1939 to 1945 |  |  |  |  |  |  |  |  | 0.024 (-0.012; 0.060) | 0.197 | 0.024 (-0.011; 0.060) | 0.183 | 0.037 (0.001; 0.073) | 0.042 | 0.038 (0.002; 0.074) | 0.041 |
| **Attrition (ref. No drop out)** |  |  |  |  |  |  |  |  |  |  |  |  |  |  |  |  |
| Drop out |  |  |  |  |  |  |  |  | 0.004 (-0.020; 0.028) | 0.72 | 0.005 (-0.019; 0.028) | 0.707 | 0.004 (-0.019; 0.028) | 0.715 | 0.005 (-0.019; 0.029) | 0.693 |
| Death |  |  |  |  |  |  |  |  | 0.552 (0.515; 0.589) | <0.001 | 0.547 (0.510; 0.584) | <0.001 | 0.548 (0.511; 0.585) | <0.001 | 0.545 (0.508; 0.582) | <0.001 |
| **Education (ref. primary)** |  |  |  |  |  |  |  |  |  |  |  |  |  |  |  |  |
| Secondary |  |  |  |  |  |  |  |  | -0.276 (-0.304; -0.248) | <0.001 | -0.273 (-0.301; -0.245) | <0.001 | -0.274 (-0.301; -0.246) | <0.001 | -0.272 (-0.299; -0.244) | <0.001 |
| Tertiary |  |  |  |  |  |  |  |  | -0.365 (-0.399; -0.332) | <0.001 | -0.363 (-0.396; -0.330) | <0.001 | -0.360 (-0.393; -0.326) | <0.001 | -0.358 (-0.391; -0.325) | <0.001 |
| **Income** |  |  |  |  |  |  |  |  |  |  |  |  |  |  |  |  |
| Fairly easily |  |  |  |  |  |  |  |  | 0.166 (0.141; 0.191) | <0.001 | 0.168 (0.143; 0.193) | <0.001 | 0.166 (0.141; 0.191) | <0.001 | 0.168 (0.143; 0.193) | <0.001 |
| With some difficulties |  |  |  |  |  |  |  |  | 0.591 (0.562; 0.621) | <0.001 | 0.592 (0.563; 0.621) | <0.001 | 0.592 (0.562; 0.621) | <0.001 | 0.592 (0.562; 0.621) | <0.001 |
| With great difficulties |  |  |  |  |  |  |  |  | 1.424 (1.387; 1.461) | <0.001 | 1.420 (1.383; 1.457) | <0.001 | 1.423 (1.386; 1.460) | <0.001 | 1.418 (1.381; 1.455) | <0.001 |
| **Level** |  |  |  |  |  |  |  |  |  |  |  |  |  |  |  |  |
| Physical activity (ref. no) |  |  |  |  |  |  |  |  |  |  |  |  |  |  |  |  |
| YES | -0.593 (-0.610; -0.577) | <0.001 | -0.411 (-0.436; -0.385) | <0.001 | -0.677 (-0.699; -0.655) | <0.001 | -0.472 (-0.509; -0.436) | <0.001 | -0.508 (-0.525; -0.492) | <0.001 | -0.338 (-0.363; -0.312) | <0.001 | -0.581 (-0.603; -0.560) | <0.001 | -0.397 (-0.433; -0.361) | <0.001 |
| Multimorbidity (ref.no) |  |  |  |  |  |  |  |  |  |  |  |  |  |  |  |  |
| YES | 0.730 (0.715; 0.746) | <0.001 | 0.951 (0.922; 0.979) | <0.001 | 0.648 (0.626; 0.669) | <0.001 | 0.868 (0.829; 0.906) | <0.001 | 0.642 (0.626; 0.657) | <0.001 | 0.847 (0.819; 0.875) | <0.001 | 0.573 (0.552; 0.594) | <0.001 | 0.771 (0.732; 0.809) | <0.001 |
| Physical activity (ref. no) x Multimorbidity (ref. no) |  |  |  |  |  |  |  |  |  |  |  |  |  |  |  |  |
| YES |  |  | -0.298 (-0.330; -0.266) | <0.001 |  |  | -0.302 (-0.346; -0.257) | <0.001 |  |  | -0.278 (-0.309; -0.247) | <0.001 |  |  | -0.272 (-0.316; -0.228) | <0.001 |
| **Rate of change** |  |  |  |  |  |  |  |  |  |  |  |  |  |  |  |  |
| Age (ref. 73 years old) | 0.377 (0.363; 0.392) | <0.001 | 0.372 (0.358; 0.387) | <0.001 | 0.457 (0.433; 0.480) | <0.001 | 0.476 (0.447; 0.505) | <0.001 | 0.325 (0.295; 0.354) | <0.001 | 0.321 (0.291; 0.351) | <0.001 | 0.385 (0.350; 0.419) | <0.001 | 0.402 (0.364; 0.440) | <0.001 |
| Age (ref. 73 years old) squared | 0.193 (0.185; 0.201) | <0.001 | 0.192 (0.183; 0.200) | <0.001 | 0.148 (0.133; 0.164) | <0.001 | 0.176 (0.156; 0.196) | <0.001 | 0.185 (0.176; 0.194) | <0.001 | 0.184 (0.175; 0.193) | <0.001 | 0.145 (0.129; 0.161) | <0.001 | 0.169 (0.149-0.189) | <0.001 |
| Age (ref. 73 years old) x Physical activity (ref. no) |  |  |  |  |  |  |  |  |  |  |  |  |  |  |  |  |
| YES |  |  |  |  | -0.113 (-0.134; -0.093) | <0.001 | -0.123 (-0.157; -0.089) | <0.001 |  |  |  |  | -0.125 (-0.145; -0.104) | <0.001 | -0.132 (-0.165; -0.098) | <0.001 |
| Age (ref. 73 years old) squared x Physical activity (ref. no) |  |  |  |  |  |  |  |  |  |  |  |  |  |  |  |  |
| YES |  |  |  |  | 0.017 (0.002; 0.031) | 0.029 | -0.013 (-0.036; 0.010) | 0.259 |  |  |  |  | 0.003 (-0.011; 0.018) | 0.646 | -0.022 (-0.044; 0.001) | 0.058 |
| Age (ref. 73 years old) x Multimorbidity (ref. no) |  |  |  |  |  |  |  |  |  |  |  |  |  |  |  |  |
| YES |  |  |  |  | -0.028 (-0.050; -0.006) | 0.012 | -0.076 (-0.108; -0.043) | <0.001 |  |  |  |  | -0.003 (-0.024-0.019) | 0.801 | -0.044 (-0.076; -0.012) | 0.006 |
| Age (ref. 73 years old) squared x Multimorbidity (ref. no) |  |  |  |  |  |  |  |  |  |  |  |  |  |  |  |  |
| YES |  |  |  |  | 0.047 (0.033; 0.062) | <0.001 | 0.017 (-0.008; 0.041) | 0.183 |  |  |  |  | 0.050 (0.036; 0.064) | <0.001 | 0.024 (0.000; 0.048) | 0.048 |
| Age (ref. 73 years old) x Physical activity (ref. no) x Multimorbidity (ref. no) |  |  |  |  |  |  |  |  |  |  |  |  |  |  |  |  |
| YES |  |  |  |  |  |  | 0.037 (-0.004; 0.079) | 0.08 |  |  |  |  |  |  | 0.031 (-0.010; 0.072) | 0.144 |
| Age (ref. 73 years old) squared x Physical activity (ref. no) x Multimorbidity (ref. no) |  |  |  |  |  |  |  |  |  |  |  |  |  |  |  |  |
| YES |  |  |  |  |  |  | 0.033 (0.003; 0.062) | 0.03 |  |  |  |  |  |  | 0.027 (-0.002; 0.056) | 0.073 |
| *R^2^*m | 0.102 |  | 0.103 |  | 0.103 |  | 0.104 |  | 0.173 |  | 0.174 |  | 0.175 |  | 0.175 |  |
| *R*^2^c | 0.516 |  | 0.515 |  | 0.515 |  | 0.515 |  | 0.522 |  | 0.522 |  | 0.522 |  | 0.522 |  |

**Note.** Minimally adjusted *level* = minimally adjusted model (i.e., age, sex, and wave) testing the independent association of multimorbidity and physical activity on the level of health indicators; Minimally adjusted *INTERACTION level* = minimally adjusted model testing the interactive association of multimorbidity and physical activity on the level of health indicators; Minimally adjusted *trajectory* = minimally adjusted model testing the independent association of multimorbidity and physical activity on the level and rate of change of the health indicators across aging; Minimally adjusted *INTERACTION trajectory* = minimally adjusted model testing the interactive association of multimorbidity and physical activity on the level and rate of change of the health indicators across aging; Models 1a, 1b, 2a, and 2b are the full results for the models tested in the main analysis.

**Table S5. Results of the mixed-effects models for well being**

|  | **Minimally adjusted *level*** | | **Minimally adjusted**  ***INTERACTION level*** | | **Minimally adjusted *trajectory*** | | **Minimally adjusted *INTERACTION trajectory*** | | **Model *1a*** | | **Model *1b*** | | **Model 2*a*** | | **Model 2b** | |
| --- | --- | --- | --- | --- | --- | --- | --- | --- | --- | --- | --- | --- | --- | --- | --- | --- |
| **Variables** | Coefficient (95% CI) | *P* value | Coefficient (95% CI) | *P* value | Coefficient (95% CI) | *P* value | Coefficient (95% CI) | *P* value | Coefficient (95% CI) | *P* value | Coefficient (95% CI) | *P* value | Coefficient (95% CI) | *P* value | Coefficient (95% CI) | *P* value |
| **Intercept** | 35.500 (35.411; 35.589) | <0.001 | 35.650 (35.551; 35.748) | <0.001 | 35.318 (35.222; 35.415) | <0.001 | 35.471 (35.358; 35.584) | <0.001 | 37.180 (36.978; 37.382) | <0.001 | 37.358 (37.152; 37.564) | <0.001 | 37.053 (36.848; 37.258) | <0.001 | 37.196 (36.983; 37.408) | <0.001 |
| **Sex (ref. Male)** | 0.544 (0.482; 0.605) | <0.001 | 0.542 (0.480; 0.603) | <0.001 | 0.540 (0.479; 0.602) | <0.001 | 0.539 (0.478; 0.601) | <0.001 | 0.335 (0.284; 0.387) | <0.001 | 0.333 (0.281; 0.384) | <0.001 | 0.329 (0.278; 0.380) | <0.001 | 0.328 (0.276; 0.379) | <0.001 |
| **Wave** | 0.119 (0.109; 0.129) | <0.001 | 0.119 (0.109; 0.129) | <0.001 | 0.119 (0.108; 0.129) | <0.001 | 0.119 (0.108; 0.129) | <0.001 | 0.103 (0.087; 0.119) | <0.001 | 0.103 (0.087; 0.119) | <0.001 | 0.100 (0.084; 0.116) | <0.001 | 0.100 (0.084; 0.116) | <0.001 |
| **Country (ref. Belgium)** |  |  |  |  |  |  |  |  |  |  |  |  |  |  |  |  |
| Austria |  |  |  |  |  |  |  |  | 1.568 (1.425; 1.710) | <0.001 | 1.565 (1.422; 1.708) | <0.001 | 1.559 (1.417; 1.702) | <0.001 | 1.558 (1.416; 1.701) | <0.001 |
| Bulgaria |  |  |  |  |  |  |  |  | -1.541 (-1.901; -1.182) | <0.001 | -1.551 (-1.910; -1.191) | <0.001 | -1.537 (-1.896; -1.178) | <0.001 | -1.545 (-1.904; -1.186) | <0.001 |
| Croatia |  |  |  |  |  |  |  |  | 0.140 (-0.071; 0.351) | 0.194 | 0.134 (-0.077; 0.345) | 0.213 | 0.143 (-0.069; 0.354) | 0.186 | 0.138 (-0.073; 0.349) | 0.2 |
| Cyprus |  |  |  |  |  |  |  |  | -0.357 (-0.837; 0.123) | 0.144 | -0.373 (-0.853; 0.107) | 0.128 | -0.343 (-0.822; 0.136) | 0.161 | -0.353 (-0.832; 0.126) | 0.148 |
| Czech Republic |  |  |  |  |  |  |  |  | -1.650 (-1.783; -1.516) | <0.001 | -1.652 (-1.785; -1.518) | <0.001 | -1.659 (-1.792; -1.525) | <0.001 | -1.660 (-1.793; -1.526) | <0.001 |
| Denmark |  |  |  |  |  |  |  |  | 2.004 (1.857; 2.151) | <0.001 | 2.007 (1.860; 2.154) | <0.001 | 2.011 (1.864; 2.158) | <0.001 | 2.013 (1.866; 2.160) | <0.001 |
| Estonia |  |  |  |  |  |  |  |  | -0.844 (-0.981; -0.706) | <0.001 | -0.844 (-0.982; -0.707) | <0.001 | -0.846 (-0.983; -0.708) | <0.001 | -0.846 (-0.983; -0.709) | <0.001 |
| Finland |  |  |  |  |  |  |  |  | -0.061 (-0.382; 0.260) | 0.708 | -0.063 (-0.384; 0.258) | 0.7 | -0.076 (-0.397; 0.245) | 0.643 | -0.076 (-0.396; 0.245) | 0.644 |
| France |  |  |  |  |  |  |  |  | 0.529 (0.392; 0.666) | <0.001 | 0.526 (0.389; 0.663) | <0.001 | 0.515 (0.378; 0.652) | <0.001 | 0.514 (0.377; 0.650) | <0.001 |
| Germany |  |  |  |  |  |  |  |  | 0.682 (0.547; 0.817) | <0.001 | 0.681 (0.547; 0.816) | <0.001 | 0.684 (0.549; 0.818) | <0.001 | 0.683 (0.548; 0.817) | <0.001 |
| Greece |  |  |  |  |  |  |  |  | -1.958 (-2.107; -1.809) | <0.001 | -1.966 (-2.115; -1.817) | <0.001 | -1.975 (-2.124; -1.826) | <0.001 | -1.981 (-2.130; -1.832) | <0.001 |
| Hungary |  |  |  |  |  |  |  |  | 0.176 (-0.028; 0.380) | 0.091 | 0.176 (-0.028; 0.380) | 0.092 | 0.181 (-0.022; 0.385) | 0.081 | 0.181 (-0.023; 0.385) | 0.082 |
| Ireland |  |  |  |  |  |  |  |  | 2.048 (1.707; 2.390) | <0.001 | 2.050 (1.708; 2.391) | <0.001 | 2.040 (1.698; 2.381) | <0.001 | 2.040 (1.699; 2.382) | <0.001 |
| Israel |  |  |  |  |  |  |  |  | -1.133 (-1.313; -0.953) | <0.001 | -1.133 (-1.313; -0.954) | <0.001 | -1.145 (-1.324; -0.965) | <0.001 | -1.145 (-1.324; -0.965) | <0.001 |
| Italy |  |  |  |  |  |  |  |  | -1.405 (-1.542; -1.268) | <0.001 | -1.415 (-1.552; -1.278) | <0.001 | -1.428 (-1.564; -1.291) | <0.001 | -1.435 (-1.572; -1.298) | <0.001 |
| Latvia |  |  |  |  |  |  |  |  | -2.071 (-2.458; -1.684) | <0.001 | -2.074 (-2.461; -1.687) | <0.001 | -2.074 (-2.460; -1.687) | <0.001 | -2.076 (-2.463; -1.690) | <0.001 |
| Lithuania |  |  |  |  |  |  |  |  | -2.701 (-2.994; -2.407) | <0.001 | -2.702 (-2.996; -2.409) | <0.001 | -2.696 (-2.990; -2.403) | <0.001 | -2.698 (-2.992; -2.405) | <0.001 |
| Luxembourg |  |  |  |  |  |  |  |  | 1.533 (1.315-1.752) | <0.001 | 1.533 (1.315-1.752) | <0.001 | 1.539 (1.320-1.758) | <0.001 | 1.539 (1.320-1.757) | <0.001 |
| Malta |  |  |  |  |  |  |  |  | -0.411 (-0.790; -0.031) | 0.034 | -0.422 (-0.802; -0.043) | 0.029 | -0.408 (-0.788; -0.029) | 0.035 | -0.418 (-0.798; -0.039) | 0.031 |
| Netherlands |  |  |  |  |  |  |  |  | 1.929 (1.780-2.078) | <0.001 | 1.933 (1.785-2.082) | <0.001 | 1.933 (1.784-2.081) | <0.001 | 1.936 (1.787-2.084) | <0.001 |
| Poland |  |  |  |  |  |  |  |  | 0.085 (-0.084-0.255) | 0.324 | 0.084 (-0.086-0.253) | 0.333 | 0.085 (-0.085-0.254) | 0.327 | 0.084 (-0.086-0.253) | 0.334 |
| Portugal |  |  |  |  |  |  |  |  | -2.308 (-2.534; -2.082) | <0.001 | -2.315 (-2.541; -2.089) | <0.001 | -2.323 (-2.549; -2.097) | <0.001 | -2.327 (-2.553; -2.101) | <0.001 |
| Romania |  |  |  |  |  |  |  |  | -1.613 (-1.950; -1.276) | <0.001 | -1.630 (-1.967; -1.293) | <0.001 | -1.643 (-1.980; -1.306) | <0.001 | -1.657 (-1.994; -1.319) | <0.001 |
| Slovakia |  |  |  |  |  |  |  |  | 2.384 (2.230-2.537) | <0.001 | 2.380 (2.227-2.534) | <0.001 | 2.382 (2.229-2.535) | <0.001 | 2.380 (2.226-2.533) | <0.001 |
| Slovenia |  |  |  |  |  |  |  |  | -0.218 (-0.354; -0.081) | 0.002 | -0.222 (-0.358; -0.085) | 0.001 | -0.231 (-0.367; -0.095) | 0.001 | -0.234 (-0.370; -0.097) | 0.001 |
| Spain |  |  |  |  |  |  |  |  | 0.925 (0.782; 1.068) | <0.001 | 0.927 (0.784; 1.069) | <0.001 | 0.912 (0.770; 1.054) | <0.001 | 0.914 (0.772; 1.057) | <0.001 |
| Sweden |  |  |  |  |  |  |  |  | 2.116 (1.959; 2.273) | <0.001 | 2.115 (1.958; 2.272) | <0.001 | 2.105 (1.948; 2.262) | <0.001 | 2.104 (1.947; 2.261) | <0.001 |
| Switzerland |  |  |  |  |  |  |  |  | 1.568 (1.425; 1.710) | <0.001 | 1.565 (1.422; 1.708) | <0.001 | 1.559 (1.417; 1.702) | <0.001 | 1.558 (1.416; 1.701) | <0.001 |
| **Birth cohort (ref. born after 1945)** |  |  |  |  |  |  |  |  |  |  |  |  |  |  |  |  |
| 1919 to 1928 |  |  |  |  |  |  |  |  | 0.522 (0.286; 0.758) | <0.001 | 0.518 (0.282; 0.754) | <0.001 | 0.485 (0.249; 0.721) | <0.001 | 0.482 (0.247; 0.718) | <0.001 |
| 1929 to 1938 |  |  |  |  |  |  |  |  | 0.049 (-0.094; 0.192) | 0.501 | 0.048 (-0.094; 0.191) | 0.507 | 0.006 (-0.137; 0.149) | 0.934 | 0.006 (-0.137; 0.149) | 0.938 |
| 1939 to 1945 |  |  |  |  |  |  |  |  | 0.017 (-0.077; 0.112) | 0.718 | 0.016 (- 0.078; 0.111) | 0.735 | -0.008 (-0.103; 0.087) | 0.871 | -0.008 (-0.103; 0.087) | 0.866 |
| **Attrition (ref. No drop out)** |  |  |  |  |  |  |  |  |  |  |  |  |  |  |  |  |
| Drop out |  |  |  |  |  |  |  |  | -0.092 (-0.154; -0.029) | 0.004 | -0.092 (-0.154; -0.029) | 0.004 | -0.091 (-0.153; -0.028) | 0.004 | -0.091 (-0.154; -0.029) | 0.004 |
| Death |  |  |  |  |  |  |  |  | -1.409 (-1.506; -1.313) | <0.001 | -1.403 (-1.500; -1.306) | <0.001 | -1.391 (-1.488; -1.295) | <0.001 | -1.388 (-1.485; -1.292) | <0.001 |
| **Education (ref. primary)** |  |  |  |  |  |  |  |  |  |  |  |  |  |  |  |  |
| Secondary |  |  |  |  |  |  |  |  | 0.748 (0.675; 0.822) | <0.001 | 0.745 (0.672; 0.818) | <0.001 | 0.740 (0.667; 0.813) | <0.001 | 0.738 (0.665; 0.811) | <0.001 |
| Tertiary |  |  |  |  |  |  |  |  | 1.202 (1.114; 1.289) | <0.001 | 1.198 (1.111; 1.286) | <0.001 | 1.188 (1.101; 1.276) | <0.001 | 1.187 (1.099; 1.274) | <0.001 |
| **Income** |  |  |  |  |  |  |  |  |  |  |  |  |  |  |  |  |
| Fairly easily |  |  |  |  |  |  |  |  | -1.577 (-1.642; -1.511) | <0.001 | -1.579 (-1.645; -1.513) | <0.001 | -1.577 (-1.643; -1.512) | <0.001 | -1.579 (-1.645; -1.513) | <0.001 |
| With some difficulties |  |  |  |  |  |  |  |  | -3.647 (-3.724; -3.570) | <0.001 | -3.647 (-3.724; -3.571) | <0.001 | -3.647 (-3.724; -3.571) | <0.001 | -3.647 (-3.724; -3.571) | <0.001 |
| With great difficulties |  |  |  |  |  |  |  |  | -6.173 (-6.270; -6.076) | <0.001 | -6.167 (-6.264; -6.070) | <0.001 | -6.175 (-6.272; -6.078) | <0.001 | -6.170 (-6.267; -6.073) | <0.001 |
| **Level** |  |  |  |  |  |  |  |  |  |  |  |  |  |  |  |  |
| Physical activity (ref. no) |  |  |  |  |  |  |  |  |  |  |  |  |  |  |  |  |
| YES | 2.046 (2.003; 2.090) | <0.001 | 1.859 (1.792; 1.926) | <0.001 | 2.274 (2.217; 2.332) | <0.001 | 2.079 (1.983; 2.174) | <0.001 | 1.700 (1.658; 1.741) | <0.001 | 1.482 (1.417; 1.546) | <0.001 | 1.928 (1.872; 1.983) | <0.001 | 1.748 (1.657; 1.839) | <0.001 |
| Multimorbidity (ref.no) |  |  |  |  |  |  |  |  |  |  |  |  |  |  |  |  |
| YES | -1.633 (-1.675; -1.591) | <0.001 | -1.860 (-1.934; -1.785) | <0.001 | -1.570 (-1.627; -1.514) | <0.001 | -1.789 (-1.892; -1.687) | <0.001 | -1.380 (-1.419; -1.340) | <0.001 | -1.643 (-1.714; -1.572) | <0.001 | -1.317 (-1.370; -1.263) | <0.001 | -1.516 (-1.613; -1.418) | <0.001 |
| Physical activity (ref. no) x Multimorbidity (ref. no) |  |  |  |  |  |  |  |  |  |  |  |  |  |  |  |  |
| YES |  |  | 0.307 (0.224; 0.390) | <0.001 |  |  | 0.297 (0.181; 0.414) | <0.001 |  |  | 0.356 (0.276; 0.436) | <0.001 |  |  | 0.269 (0.158; 0.380) | <0.001 |
| **Rate of change** |  |  |  |  |  |  |  |  |  |  |  |  |  |  |  |  |
| Age (ref. 73 years old) | -1.348 (-1.389; -1.308) | <0.001 | -1.343 (-1.384; -1.303) | <0.001 | -1.491 (-1.554; -1.429) | <0.001 | -1.516 (-1.591; -1.440) | <0.001 | -1.223 (-1.300; -1.146) | <0.001 | -1.218 (-1.295; -1.141) | <0.001 | -1.369 (-1.457; -1.280) | <0.001 | -1.416 (-1.514; -1.319) | <0.001 |
| Age (ref. 73 years old) squared | -0.522 (-0.544; -0.499) | <0.001 | -0.520 (-0.542; -0.498) | <0.001 | -0.420 (-0.460; -0.379) | <0.001 | -0.466 (-0.518; -0.414) | <0.001 | -0.501 (-0.524; -0.477) | <0.001 | -0.499 (-0.523; -0.476) | <0.001 | -0.403 (-0.443; -0.363) | <0.001 | -0.442 (-0.493; -0.392) | <0.001 |
| Age (ref. 73 years old) x Physical activity (ref. no) |  |  |  |  |  |  |  |  |  |  |  |  |  |  |  |  |
| YES |  |  |  |  | 0.261 (0.207; 0.315) | <0.001 | 0.286 (0.198; 0.373) | <0.001 |  |  |  |  | 0.336 (0.284; 0.388) | <0.001 | 0.394 (0.310; 0.479) | <0.001 |
| Age (ref. 73 years old) squared x Physical activity (ref. no) |  |  |  |  |  |  |  |  |  |  |  |  |  |  |  |  |
| YES |  |  |  |  | -0.071 (-0.110; -0.032) | <0.001 | -0.014 (-0.074; 0.045) | 0.643 |  |  |  |  | -0.038 (-0.075; -0.001) | 0.047 | 0.013 (-0.044; 0.070) | 0.66 |
| Age (ref. 73 years old) x Multimorbidity (ref. no) |  |  |  |  |  |  |  |  |  |  |  |  |  |  |  |  |
| YES |  |  |  |  | 0.004 (-0.053; 0.060) | 0.901 | 0.051 (-0.033; 0.135) | 0.23 |  |  |  |  | -0.036 (-0.090; 0.018) | 0.196 | 0.049 (-0.031; 0.130) | 0.232 |
| Age (ref. 73 years old) squared x Multimorbidity (ref. no) |  |  |  |  |  |  |  |  |  |  |  |  |  |  |  |  |
| YES |  |  |  |  | -0.045 (-0.083; -0.007) | 0.02 | 0.022 (-0.042; 0.085) | 0.507 |  |  |  |  | -0.065 (-0.101; -0.029) | <0.001 | -0.017 (-0.078; 0.044) | 0.583 |
| Age (ref. 73 years old) x Physical activity (ref. no) x Multimorbidity (ref. no) |  |  |  |  |  |  |  |  |  |  |  |  |  |  |  |  |
| YES |  |  |  |  |  |  | -0.054 (-0.161; 0.054) | 0.327 |  |  |  |  |  |  | -0.109 (-0.213; -0.006) | 0.039 |
| Age (ref. 73 years old) squared x Physical activity (ref. no) x Multimorbidity (ref. no) |  |  |  |  |  |  |  |  |  |  |  |  |  |  |  |  |
| YES |  |  |  |  |  |  | -0.087 (-0.164; -0.010) | 0.027 |  |  |  |  |  |  | -0.067 (-0.141; 0.007) | 0.075 |
| *R^2^*m | 0.087 |  | 0.087 |  | 0.087 |  | 0.088 |  | 0.329 |  | 0.330 |  | 0.331 |  | 0.331 |  |
| *R*^2^c | 0.627 |  | 0.627 |  | 0.627 |  | 0.626 |  | 0.637 |  | 0.636 |  | 0.636 |  | 0.636 |  |

**Note.** Minimally adjusted *level* = minimally adjusted model (i.e., age, sex, and wave) testing the independent association of multimorbidity and physical activity on the level of health indicators; Minimally adjusted *INTERACTION level* = minimally adjusted model testing the interactive association of multimorbidity and physical activity on the level of health indicators; Minimally adjusted *trajectory* = minimally adjusted model testing the independent association of multimorbidity and physical activity on the level and rate of change of the health indicators across aging; Minimally adjusted *INTERACTION trajectory* = minimally adjusted model testing the interactive association of multimorbidity and physical activity on the level and rate of change of the health indicators across aging; Models 1a, 1b, 2a, and 2b are the full results for the models tested in the main analysis.

**Table S6. Results of the mixed-effects models for self-rated health**

|  | **Minimally adjusted *level*** | | **Minimally adjusted**  ***INTERACTION level*** | | **Minimally adjusted *trajectory*** | | **Minimally adjusted *INTERACTION trajectory*** | | **Model *1a*** | | **Model *1b*** | | **Model 2*a*** | | **Model 2b** | |
| --- | --- | --- | --- | --- | --- | --- | --- | --- | --- | --- | --- | --- | --- | --- | --- | --- |
| **Variables** | Coefficient (95% CI) | *P* value | Coefficient (95% CI) | *P* value | Coefficient (95% CI) | *P* value | Coefficient (95% CI) | *P* value | Coefficient (95% CI) | *P* value | Coefficient (95% CI) | *P* value | Coefficient (95% CI) | *P* value | Coefficient (95% CI) | *P* value |
| **Intercept** | 2.767 (2.754; 2.780) | <0.001 | 2.781 (2.766; 2.795) | <0.001 | 2.714 (2.700; 2.729) | <0.001 | 2.727 (2.710; 2.744) | <0.001 | 3.027 (2.994; 3.060) | <0.001 | 3.036 (3.003; 3.070) | <0.001 | 2.995 (2.962; 3.029) | <0.001 | 3.004 (2.969; 3.038) | <0.001 |
| **Sex (ref. Male)** | 0.045 (0.036; 0.054) | <0.001 | 0.044 (0.035; 0.054) | <0.001 | 0.045 (0.036; 0.054) | <0.001 | 0.045 (0.036; 0.054) | <0.001 | 0.021 (0.013; 0.029) | <0.001 | 0.021 (0.013; 0.029) | <0.001 | 0.022 (0.013; 0.030) | <0.001 | 0.022 (0.013; 0.030) | <0.001 |
| **Wave** | -0.005 (-0.006; -0.003) | <0.001 | -0.005 (-0.006; -0.003) | <0.001 | -0.005 (-0.006; -0.003) | <0.001 | -0.005 (-0.006; -0.003) | <0.001 | -0.011 (-0.014; -0.008) | <0.001 | -0.011 (-0.014; -0.008) | <0.001 | -0.012 (-0.014; -0.009) | <0.001 | -0.012 (-0.014; -0.009) | <0.001 |
| **Country (ref. Belgium)** |  |  |  |  |  |  |  |  |  |  |  |  |  |  |  |  |
| Austria |  |  |  |  |  |  |  |  | -0.050 (-0.073; -0.027) | <0.001 | -0.050 (-0.073; -0.027) | <0.001 | -0.051 (-0.074; -0.028) | <0.001 | -0.051 (-0.074; -0.028) | <0.001 |
| Bulgaria |  |  |  |  |  |  |  |  | -0.048 (-0.108-0.011) | 0.112 | -0.049 (-0.108-0.011) | 0.109 | -0.052 (-0.111-0.008) | 0.09 | -0.053 (-0.112-0.007) | 0.082 |
| Croatia |  |  |  |  |  |  |  |  | -0.167 (-0.202; -0.132) | <0.001 | -0.168 (-0.203; -0.133) | <0.001 | -0.168 (-0.202; -0.133) | <0.001 | -0.168 (-0.203; -0.133) | <0.001 |
| Cyprus |  |  |  |  |  |  |  |  | 0.150 (0.074-0.226) | <0.001 | 0.149 (0.073-0.225) | <0.001 | 0.151 (0.075-0.227) | <0.001 | 0.151 (0.075-0.227) | <0.001 |
| Czech Republic |  |  |  |  |  |  |  |  | -0.239 (-0.260; -0.217) | <0.001 | -0.239 (-0.260; -0.217) | <0.001 | -0.241 (-0.263; -0.220) | <0.001 | -0.242 (-0.263; -0.220) | <0.001 |
| Denmark |  |  |  |  |  |  |  |  | 0.227 (0.203-0.251) | <0.001 | 0.227 (0.203-0.251) | <0.001 | 0.227 (0.203-0.250) | <0.001 | 0.227 (0.203-0.251) | <0.001 |
| Estonia |  |  |  |  |  |  |  |  | -0.700 (-0.722; -0.678) | <0.001 | -0.700 (-0.722; -0.678) | <0.001 | -0.701 (-0.723; -0.679) | <0.001 | -0.701 (-0.723; -0.679) | <0.001 |
| Finland |  |  |  |  |  |  |  |  | -0.227 (-0.280; -0.175) | <0.001 | -0.227 (-0.280; -0.175) | <0.001 | -0.228 (-0.281; -0.176) | <0.001 | -0.228 (-0.281; -0.176) | <0.001 |
| France |  |  |  |  |  |  |  |  | -0.140 (-0.162; -0.118) | <0.001 | -0.140 (-0.162; -0.118) | <0.001 | -0.142 (-0.164; -0.121) | <0.001 | -0.143 (-0.164; -0.121) | <0.001 |
| Germany |  |  |  |  |  |  |  |  | -0.351 (-0.373; -0.329) | <0.001 | -0.351 (-0.373; -0.329) | <0.001 | -0.352 (-0.374; -0.330) | <0.001 | -0.352 (-0.374; -0.330) | <0.001 |
| Greece |  |  |  |  |  |  |  |  | 0.299 (0.275; 0.324) | <0.001 | 0.299 (0.275; 0.323) | <0.001 | 0.293 (0.269; 0.317) | <0.001 | 0.291 (0.267; 0.316) | <0.001 |
| Hungary |  |  |  |  |  |  |  |  | -0.391 (-0.424; -0.357) | <0.001 | -0.391 (-0.424; -0.357) | <0.001 | -0.388 (-0.422; -0.354) | <0.001 | -0.388 (-0.422; -0.354) | <0.001 |
| Ireland |  |  |  |  |  |  |  |  | 0.365 (0.307; 0.422) | <0.001 | 0.365 (0.307; 0.422) | <0.001 | 0.363 (0.305; 0.420) | <0.001 | 0.362 (0.305; 0.419) | <0.001 |
| Israel |  |  |  |  |  |  |  |  | -0.078 (-0.106; -0.050) | <0.001 | -0.078 (-0.106; -0.050) | <0.001 | -0.082 (-0.110; -0.054) | <0.001 | -0.082 (-0.110; -0.054) | <0.001 |
| Italy |  |  |  |  |  |  |  |  | -0.026 (-0.049; -0.004) | 0.019 | -0.027 (-0.049; -0.005) | 0.017 | -0.032 (-0.054; -0.010) | 0.005 | -0.033 (-0.055; -0.011) | 0.004 |
| Latvia |  |  |  |  |  |  |  |  | -0.770 (-0.834; -0.706) | <0.001 | -0.770 (-0.834; -0.707) | <0.001 | -0.773 (-0.836; -0.709) | <0.001 | -0.773 (-0.837; -0.709) | <0.001 |
| Lithuania |  |  |  |  |  |  |  |  | -0.488 (-0.536; -0.439) | <0.001 | -0.488 (-0.536; -0.439) | <0.001 | -0.488 (-0.536; -0.440) | <0.001 | -0.489 (-0.537; -0.441) | <0.001 |
| Luxembourg |  |  |  |  |  |  |  |  | -0.101 (-0.137; -0.065) | <0.001 | -0.101 (-0.137; -0.065) | <0.001 | -0.100 (-0.136; -0.065) | <0.001 | -0.101 (-0.136; -0.065) | <0.001 |
| Malta |  |  |  |  |  |  |  |  | -0.045 (-0.108; 0.017) | 0.157 | -0.046 (-0.109; 0.017) | 0.151 | -0.046 (-0.109; 0.017) | 0.151 | -0.047 (-0.110; 0.016) | 0.144 |
| Netherlands |  |  |  |  |  |  |  |  | -0.103 (-0.127; -0.079) | <0.001 | -0.103 (-0.127; -0.079) | <0.001 | -0.104 (-0.128; -0.080) | <0.001 | -0.104 (-0.128; -0.080) | <0.001 |
| Poland |  |  |  |  |  |  |  |  | -0.454 (-0.482; -0.426) | <0.001 | -0.454 (-0.482; -0.426) | <0.001 | -0.454 (-0.482; -0.427) | <0.001 | -0.455 (-0.483; -0.427) | <0.001 |
| Portugal |  |  |  |  |  |  |  |  | -0.381 (-0.418; -0.344) | <0.001 | -0.381 (-0.418; -0.344) | <0.001 | -0.381 (-0.418; -0.344) | <0.001 | -0.382 (-0.419; -0.345) | <0.001 |
| Romania |  |  |  |  |  |  |  |  | -0.339 (-0.390; -0.288) | <0.001 | -0.339 (-0.390; -0.288) | <0.001 | -0.339 (-0.390; -0.287) | <0.001 | -0.339 (-0.390; -0.288) | <0.001 |
| Slovakia |  |  |  |  |  |  |  |  | 0.082 (0.025; 0.140) | 0.005 | 0.082 (0.024; 0.139) | 0.005 | 0.071 (0.014; 0.129) | 0.015 | 0.069 (0.012; 0.127) | 0.018 |
| Slovenia |  |  |  |  |  |  |  |  | -0.199 (-0.223; -0.174) | <0.001 | -0.199 (-0.224; -0.174) | <0.001 | -0.200 (-0.225; -0.175) | <0.001 | -0.200 (-0.225; -0.175) | <0.001 |
| Spain |  |  |  |  |  |  |  |  | -0.141 (-0.163; -0.119) | <0.001 | -0.141 (-0.163; -0.120) | <0.001 | -0.146 (-0.167; -0.124) | <0.001 | -0.146 (-0.168; -0.124) | <0.001 |
| Sweden |  |  |  |  |  |  |  |  | 0.168 (0.145; 0.191) | <0.001 | 0.168 (0.145; 0.191) | <0.001 | 0.166 (0.143; 0.189) | <0.001 | 0.166 (0.143; 0.189) | <0.001 |
| Switzerland |  |  |  |  |  |  |  |  | 0.145 (0.119; 0.170) | <0.001 | 0.144 (0.119; 0.170) | <0.001 | 0.141 (0.116; 0.167) | <0.001 | 0.141 (0.115; 0.166) | <0.001 |
| **Birth cohort (ref. born after 1945)** |  |  |  |  |  |  |  |  |  |  |  |  |  |  |  |  |
| 1919 to 1928 |  |  |  |  |  |  |  |  | 0.162 (0.126; 0.198) | <0.001 | 0.162 (0.125; 0.198) | <0.001 | 0.149 (0.112; 0.185) | <0.001 | 0.148 (0.112; 0.184) | <0.001 |
| 1929 to 1938 |  |  |  |  |  |  |  |  | 0.025 (0.002; 0.048) | 0.03 | 0.025 (0.002; 0.048) | 0.03 | 0.012 (-0.011; 0.035) | 0.315 | 0.011 (-0.012; 0.034) | 0.338 |
| 1939 to 1945 |  |  |  |  |  |  |  |  | 0.004 (-0.012; 0.019) | 0.629 | 0.004 (-0.012; 0.019) | 0.634 | -0.002 (-0.017; 0.014) | 0.816 | - 0.002 (-0.018; 0.013) | 0.788 |
| **Attrition (ref. No drop out)** |  |  |  |  |  |  |  |  |  |  |  |  |  |  |  |  |
| Drop out |  |  |  |  |  |  |  |  | -0.063 (-0.073; -0.053) | <0.001 | -0.063 (-0.073; -0.053) | <0.001 | -0.063 (-0.073; -0.053) | <0.001 | -0.063 (-0.073; -0.053) | <0.001 |
| Death |  |  |  |  |  |  |  |  | -0.362 (-0.376; -0.347) | <0.001 | -0.361 (-0.376; -0.347) | <0.001 | -0.361 (-0.376; -0.347) | <0.001 | -0.361 (-0.376; -0.346) | <0.001 |
| **Education (ref. primary)** |  |  |  |  |  |  |  |  |  |  |  |  |  |  |  |  |
| Secondary |  |  |  |  |  |  |  |  | 0.142 (0.130; 0.153) | <0.001 | 0.142 (0.130; 0.153) | <0.001 | 0.141 (0.130; 0.153) | <0.001 | 0.141 (0.129; 0.152) | <0.001 |
| Tertiary |  |  |  |  |  |  |  |  | 0.327 (0.313; 0.341) | <0.001 | 0.327 (0.313; 0.341) | <0.001 | 0.324 (0.310; 0.338) | <0.001 | 0.324 (0.310; 0.338) | <0.001 |
| **Income** |  |  |  |  |  |  |  |  |  |  |  |  |  |  |  |  |
| Fairly easily |  |  |  |  |  |  |  |  | -0.151 (-0.161; -0.140) | <0.001 | -0.151 (-0.162; -0.140) | <0.001 | -0.151 (-0.161; -0.140) | <0.001 | -0.151 (-0.161; -0.140) | <0.001 |
| With some difficulties |  |  |  |  |  |  |  |  | -0.293 (-0.306; -0.281) | <0.001 | -0.293 (-0.306; -0.281) | <0.001 | -0.293 (-0.305; -0.280) | <0.001 | -0.293 (-0.305; -0.280) | <0.001 |
| With great difficulties |  |  |  |  |  |  |  |  | -0.507 (-0.523; -0.492) | <0.001 | -0.507 (-0.522; -0.491) | <0.001 | -0.505 (-0.521; -0.490) | <0.001 | -0.505 (-0.520; -0.489) | <0.001 |
| **Level** |  |  |  |  |  |  |  |  |  |  |  |  |  |  |  |  |
| Physical activity (ref. no) |  |  |  |  |  |  |  |  |  |  |  |  |  |  |  |  |
| YES | 0.312 (0.305; 0.319) | <0.001 | 0.295 (0.284; 0.306) | <0.001 | 0.348 (0.339; 0.358) | <0.001 | 0.333 (0.317; 0.348) | <0.001 | 0.274 (0.268; 0.281) | <0.001 | 0.263 (0.253; 0.274) | <0.001 | 0.301 (0.292; 0.310) | <0.001 | 0.292 (0.277; 0.307) | <0.001 |
| Multimorbidity (ref.no) |  |  |  |  |  |  |  |  |  |  |  |  |  |  |  |  |
| YES | - 0.528 (-0.535; -0.521) | <0.001 | -0.548 (-0.560; -0.537) | <0.001 | -0.481 (-0.490; -0.472) | <0.001 | -0.499 (-0.515; -0.483) | <0.001 | -0.498 (-0.504; -0.491) | <0.001 | -0.511 (-0.523; -0.500) | <0.001 | -0.451 (-0.460; -0.442) | <0.001 | -0.461 (-0.477; -0.445) | <0.001 |
| Physical activity (ref. no) x Multimorbidity (ref. no) |  |  |  |  |  |  |  |  |  |  |  |  |  |  |  |  |
| YES |  |  | 0.028 (0.015; 0.041) | <0.001 |  |  | 0.023 (0.005; 0.042) | 0.014 |  |  | 0.018 (0.005; 0.031) | 0.005 |  |  | 0.012 (-0.006; 0.030) | 0.179 |
| **Rate of change** |  |  |  |  |  |  |  |  |  |  |  |  |  |  |  |  |
| Age (ref. 73 years old) | -0.248 (-0.254; -0.243) | <0.001 | -0.248 (-0.253; -0.243) | <0.001 | -0.322 (-0.331; -0.313) | <0.001 | -0.334 (-0.345; -0.323) | <0.001 | -0.224 (-0.236; -0.212) | <0.001 | -0.224 (-0.236; -0.212) | <0.001 | -0.287 (-0.301; -0.273) | <0.001 | -0.299 (-0.314; -0.284) | <0.001 |
| Age (ref. 73 years old) squared | -0.030 (-0.033; -0.026) | <0.001 | -0.029 (-0.033; -0.026) | <0.001 | -0.021 (-0.027; -0.015) | <0.001 | -0.025 (-0.033; -0.017) | <0.001 | -0.030 (-0.034; -0.027) | <0.001 | -0.030 (-0.034; -0.027) | <0.001 | -0.026 (-0.033; -0.020) | <0.001 | -0.028 (-0.036; -0.021) | <0.001 |
| Age (ref. 73 years old) x Physical activity (ref. no) |  |  |  |  |  |  |  |  |  |  |  |  |  |  |  |  |
| YES |  |  |  |  | 0.054 (0.045; 0.062) | <0.001 | 0.071 (0.058; 0.085) | <0.001 |  |  |  |  | 0.047 (0.039; 0.054) | <0.001 | 0.065 (0.052; 0.078) | <0.001 |
| Age (ref. 73 years old) squared x Physical activity (ref. no) |  |  |  |  |  |  |  |  |  |  |  |  |  |  |  |  |
| YES |  |  |  |  | -0.008 (-0.014; -0.002) | 0.011 | -0.002 (-0.011; 0.008) | 0.72 |  |  |  |  | -0.002 (-0.008; 0.004) | 0.493 | 0.002 (-0.007; 0.011) | 0.673 |
| Age (ref. 73 years old) x Multimorbidity (ref. no) |  |  |  |  |  |  |  |  |  |  |  |  |  |  |  |  |
| YES |  |  |  |  | 0.071 (0.063; 0.080) | <0.001 | 0.091 (0.079; 0.104) | <0.001 |  |  |  |  | 0.069 (0.060; 0.077) | <0.001 | 0.089 (0.077; 0.101) | <0.001 |
| Age (ref. 73 years old) squared x Multimorbidity (ref. no) |  |  |  |  |  |  |  |  |  |  |  |  |  |  |  |  |
| YES |  |  |  |  | -0.003 (-0.009; 0.003) | 0.279 | -0.000 (-0.010; 0.009) | 0.928 |  |  |  |  | -0.004 (-0.010; 0.002) | 0.156 | -0.004 (-0.014; 0.005) | 0.381 |
| Age (ref. 73 years old) x Physical activity (ref. no) x Multimorbidity (ref. no) |  |  |  |  |  |  |  |  |  |  |  |  |  |  |  |  |
| YES |  |  |  |  |  |  | -0.030 (-0.047; -0.014) | <0.001 |  |  |  |  |  |  | -0.031 (-0.047; -0.015) | <0.001 |
| Age (ref. 73 years old) squared x Physical activity (ref. no) x Multimorbidity (ref. no) |  |  |  |  |  |  |  |  |  |  |  |  |  |  |  |  |
| YES |  |  |  |  |  |  | -0.006 (-0.018; 0.006) | 0.302 |  |  |  |  |  |  | -0.003 (-0.014; 0.009) | 0.656 |
| *R^2^*m | 0.172 |  | 0.172 |  | 0.175 |  | 0.175 |  | 0.313 |  | 0.313 |  | 0.315 |  | 0.313 |  |
| *R*^2^c | 0.582 |  | 0.582 |  | 0.581 |  | 0.581 |  | 0.597 |  | 0.597 |  | 0.597 |  | 0.597 |  |

**Note.** Minimally adjusted *level* = minimally adjusted model (i.e., age, sex, and wave) testing the independent association of multimorbidity and physical activity on the level of health indicators; Minimally adjusted *INTERACTION level* = minimally adjusted model testing the interactive association of multimorbidity and physical activity on the level of health indicators; Minimally adjusted *trajectory* = minimally adjusted model testing the independent association of multimorbidity and physical activity on the level and rate of change of the health indicators across aging; Minimally adjusted *INTERACTION trajectory* = minimally adjusted model testing the interactive association of multimorbidity and physical activity on the level and rate of change of the health indicators across aging; Models 1a, 1b, 2a, and 2b are the full results for the models tested in the main analysis.
